# Chemical Constituents from the Rhizomes of *Smilax glabra* and Their Antimicrobial Activity

**DOI:** 10.3390/molecules18055265

**Published:** 2013-05-08

**Authors:** Shuo Xu, Ming-Ying Shang, Guang-Xue Liu, Feng Xu, Xuan Wang, Cheng-Chao Shou, Shao-Qing Cai

**Affiliations:** 1 State Key Laboratory of Natural and Biomimetic Drugs, School of Pharmaceutical Sciences, Peking University, No. 38, Xueyuan Road, Beijing 100191, China; E-Mails: jessica06250917@126.com (S.X.); guangxl@bjmu.edu.cn (G.-X.L.); xufeng_pharm@163.com (F.X.); xuanwang6818@bjmu.edu.cn (X.W.); 2 Key Laboratory of Carcinogenesis and Translational Research (Ministry of Education), Department of Biochemistry and Molecular Biology, Peking University Cancer Hospital & Institute, No. 52 Fucheng Road, Beijing 100142, China; E-Mail: cshou@vip.sina.com

**Keywords:** *Smilax glabra*, phenylpropanoid-substituted epicatechin, homoisoflavanone, stilbenes, antimicrobial activity

## Abstract

Six new phenolic compounds, named smiglabrone A (**1**), smiglabrone B (**2**), smilachromanone (**3**), smiglastilbene (**4**), smiglactone (**5**), smiglabrol (**6**), together with fifty-seven known ones **7**–**63**were isolated from the rhizomes of *Smilax glabra*. Their structures were elucidated on the basis of extensive spectroscopic analyses, as well as by comparison with literature data. Twenty-seven of these compounds were obtained from and identified in the genus *Smilax* for the first time. The absolute configuration of (2*S*)-1,2-*O*-di-*trans*-*p*-coumaroylglycerol (**43**) was determined for the first time using the exciton-coupled circular dichroism (ECCD) method. Thirty isolated compounds were evaluated for their antimicrobial activity against three Gram-negative bacteria, three Gram-positive bacteria and one fungus, and the corresponding structure-activity relationships were also discussed. Eighteen compounds were found to be antimicrobial against the microorganisms tested and the minimum inhibitory concentrations (MIC) were in the range of 0.0794–3.09 mM. Among them, compound **1** showed antimicrobial activity against *Canidia albicans* with MIC value of 0.146 mM, which was stronger than cinchonain Ia with an MIC of 0.332 mM. Compounds **3** and **4** exhibited inhibitory activity against *Staphylococcus aureus* with MIC values of 0.303 and 0.205 mM, respectively. The results indicated that these antimicrobial constituents of this crude drug might be responsible for its clinical antimicrobial effect.

## 1. Introduction

The genus *Smilax* (Liliaceae) includes about 300 species and is widely distributed in tropical and temperate regions throughout the World, especially in East Asia and North America [1]. Many of them have been long used as medicinal herbs, especially in China as Traditional Chinese Medicines (TCM) [1]. As one of the most popular and important TCM in the genus, *Smilax glabra* Roxb., is an evergreen vine widely distributed in southern China [2]. The rhizomes of *S. glabra*, known as Tufuling in China, are used as a TCM for detoxication, clearing heat, relieving dampness and easing joint movement [2,3]. Modern pharmacological research showed that the *S. glabra* extracts possessed anti-inflammatory, immunomodulatory, protective against hepatocyte damage and anti-tumor effects [4,5]. The 95% ethanol and ethyl acetate extracts of this herb were reported to show antibacterial activity *in vitro* using the K-B paper dispersion and the broth dilution methods [6]. Previous phytochemical investigations have shown that the main constituents in the rhizomes of *S. glabra* include flavonoids, phenylpropanoids and phenolic acids [2,5]. Astilbin was thought to be the main bioactive constituent and reported to have antibacterial, antitumor, anti-inflammatory, selective immunosuppressive and antioxidant properties [7,8]. The four other constituents, including three stereoisomers of astilbin named neoastilbin, isoastilbin and neoisoastilbin, as well as their aglycon taxifolin, also displayed antibacterial and antitumor activities [8].

**Figure 1:**
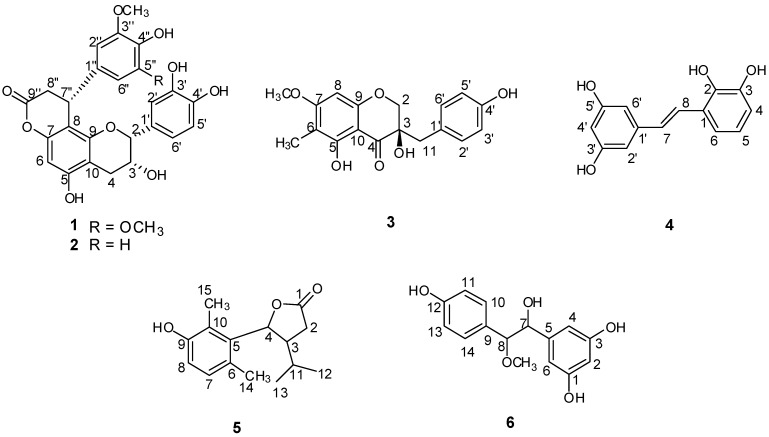
Chemical structures of compounds **1**–**6**.

**Figure 2:**
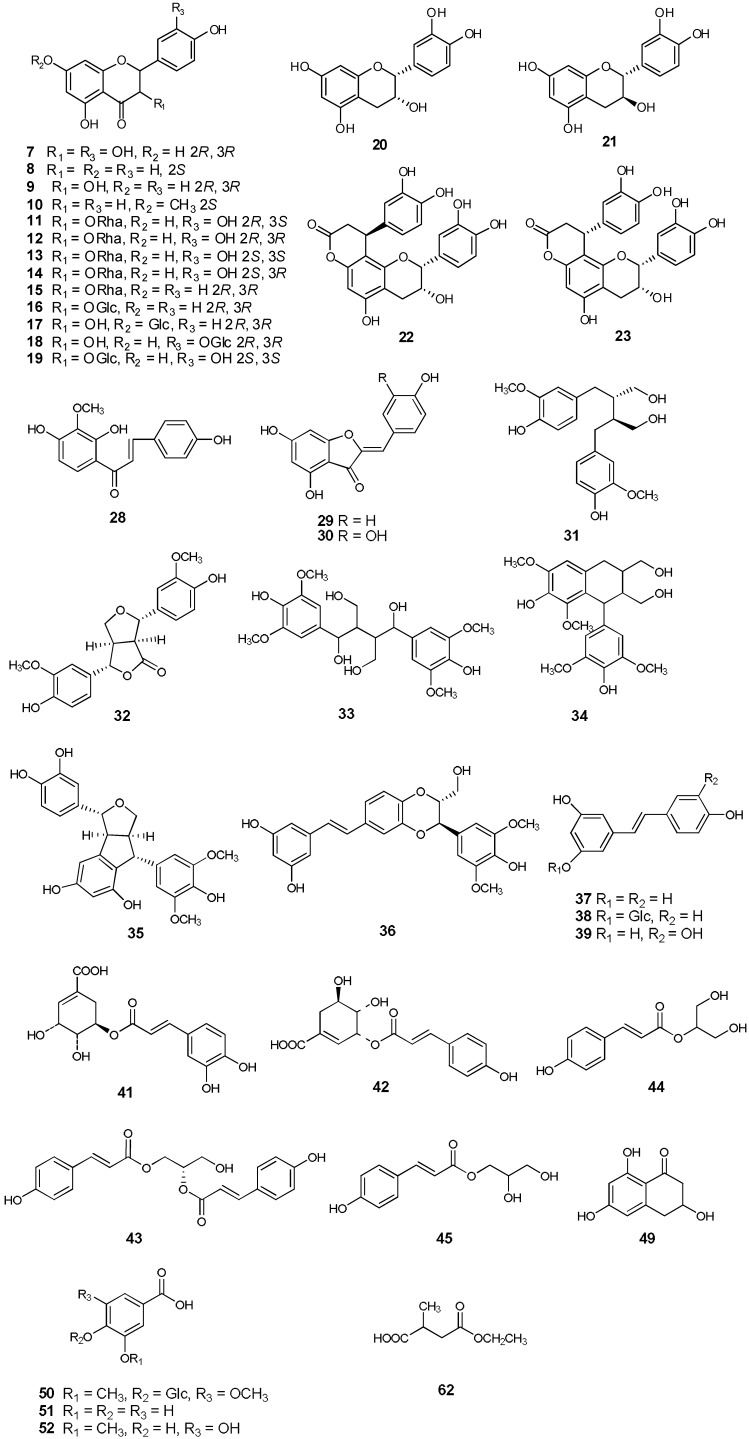
Chemical structures of compounds **7**–**23**, **28**–**45**, **49**–**52** and **62**.

It have been demonstrated that the rhizomes of *S. glabra* can be used as a TCM for numerous conditions, including acute bacterial dysentery, colds, cancer, nephritis, mercury poisoning, rheumatoid arthritis, colitis and skin disorders [2,3,9]. Many of the conditions are of infective etiology, which may point to the antimicrobial efficacy of this crude drug. Previous pharmacological investigations indicated that the rhizomes of *S. glabra* had antibacterial activity. However, except for several flavanones mentioned above, little is known about the chemical constituents that contribute to its antimicrobial activity. To clarify the structures and bioactivities of uncharacterized constituents, we carried out further chemical investigation on the rhizomes of *S. glabra*. A comprehensive chromatographic separation of the bioactive ethyl acetate and *n*-butanol fractions resulted in the isolation of sixty-three constituents (Figure 1, Figure 2), including six new compounds **1**–**6**. Compounds **1** and **2** are two new phenylpropanoid-substituted epicatechins and their structures are closely related to cinchonain Ia, that possesses antioxidant [10], antifungal and antiviral activities [11]. Compounds **3**, **4**, **5** and **6** are the corresponding homoisoflavanone, stilbene, lactone and benzene ring derivatives, respectively. Their structures were elucidated based on 1D and 2D NMR, CD, IR, and MS spectroscopic data, along with comparison with literature data. In an effort to determine the active principles of *S. glabra*, and as a part of our research for new source of antimicrobial compounds, *in vitro* tests were performed to determine the inhibitory activity of extracts and compounds from this species against three Gram-negative bacteria: *Escherichia coli*, *Pseudomonas aeruginosa* PA01 and *Kiebsiella pneumonia* (clinical isolate), three Gram-positive bacteria: methicillin-resistant *Staphylococcus aureus* (clinical isolate), *Staphylococcus aureus* ATCC6538 and *Enterococcus faecalis* and one fungus, *Canidia albicans* SC5314. In this paper, we report the isolation and structure elucidation of the new compounds and the antimicrobial activity of most of the isolated constituents.

## 2. Results and Discussion

### 2.1. Structural Elucidation of the New Compounds

**Figure 3:**
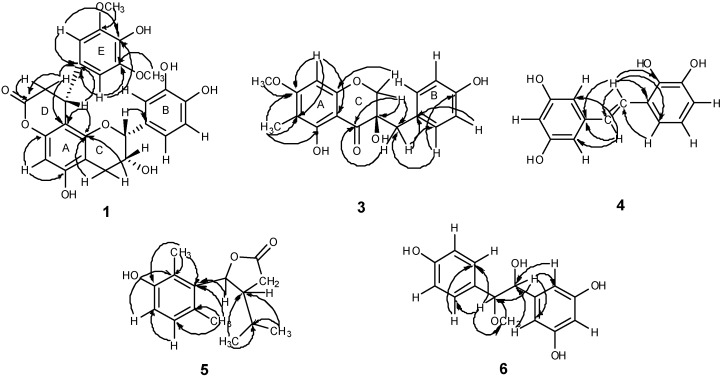
Main HMBC correlations of compounds **1**, **3**–**6**.

Compound **1 **(Figure 1) was obtained as a pale yellow solid. The molecular formula of compound **1**, C_26_H_24_O_10_, was concluded from its HRESIMS, which showed a quasi-molecular ion peak at *m/z* 495.1299 [M-H]^−^ (calcd. for C_26_H_23_O_10_ 495.1291) in the negative ion mode. Its IR spectrum showed absoptions due to hydroxyl (3,452 cm^−1^) and carbonyl (1,751 cm^−1^) groups. In the UV spectrum, absorption maxima at 202, 230 and 280 nm were observed. The ^1^H-NMR and ^13^C-NMR spectra (Table 1) showed one ABX coupling system at *δ* 7.06 (1H, d, *J* = 1.9 Hz), *δ* 6.79 (1H, d, *J* = 8.1 Hz) and *δ* 6.84 (1H, dd, *J* = 8.1, 1.9 Hz), two geminal coupling proton signals at *δ* 2.92 (1H, dd, *J* = 17.2, 4.5 Hz) and *δ* 2.85 (1H, dd, *J* = 17.2, 1.7 Hz), and three aliphatic carbon signals at *δ* 79.8 (C-2), *δ* 66.8 (C-3), *δ* 29.8 (C-4). These findings revealed the presence of an epicatechin unit. In the ^1^H-NMR spectrum of **1**, an aromatic proton singlet at *δ* 6.20 was observed, which suggested substitution at the 6- or 8-position of the A-ring. The presence of one symmetrical 1,3,4,5-tetrasubstituted phenyl group was deduced from the signal at *δ* 6.43 (2H, s). The ^1^H-NMR spectrum also indicated the presence of two methoxy groups at *δ* 3.59 (6H, s), a methine proton at *δ* 4.60 (1H, br.d, *J* = 6.3 Hz), and the methylene protons at *δ* 3.12 (1H, dd, *J* = 15.9, 7.5 Hz) and *δ* 2.91 (1H, dd, *J* = 15.9, 1.5 Hz). In addition, resonances attributable to a methine carbon at *δ* 36.2 and a methylene carbon at *δ* 37.5 were observed. The HMBC spectrum (Figure 3) showed correlations from H-2′′ to C-7′′ (*δ* 36.2), H-7′′ to C-1′′ (*δ* 135.2) and H-8′′ to C-9′′ (*δ* 171.0), indicating the presence of a phenylpropanoid unit. In the HMBC spectrum, correlations of H-8′′ with C-8 (*δ* 106.2), C-1′′ (*δ* 135.2) and C-9′′ (*δ* 171.0) indicated that the phenylpropanoid unit was attached to the C-8 position of the epicatechin, which was further confirmed by the diagnostic ^13^C-NMR resonances for the C-10, C-6 and C-8 carbons at *δ* 105.4, 96.3 and 106.1. It has been established that in the NMR spectra the position of the C-10 chemical shift is distinctive for the location of the lactone function in the A-ring. The C-8 location has the C-10 chemical shift downfield (*δ* 105) relative to the C-6 regioisomer (*δ* 100) [12]. For compound **1**, the C-10 resonance was in accordance with those of C-8 phenylpropanoid-substituted epicatechins. Furthermore, the ^13^C-NMR resonance at *δ* 171.0 (C-9′′) indicated that the phenylpropanoid unit was fused to the OH group at C-7 of the A-ring of the epicatechin unit through an ester linkage. The HMBC correlation between the methoxy protons at *δ* 3.59 (6H, s) and *δ* 149.2 (C-3′′, 5′′) confirmed the methoxy groups were linked to C-3′′, 5′′. Thus, compound **1** was very similar to cinchonain Ia, previously isolated from the bark of *Cinchona succirubra*, but with additional *O*-methyl groups [13]. The absolute configuration of C-7′′ was defined based on the CD spectrum, which showed negative Cotton effects at 235 and 283 nm and a positive Cotton effect at 259 nm. Hence, the absolute configuration of C-7′′ was determined to be *R* [13]. Consequently, the structure of **1** was elucidated as epicatechin-(7,8-bc)-4*α*-(4-hydroxy-3,5-dimethoxyphenyl)-dihydro-2(3*H*)-pyranone, and was trivially named smiglabrone A.

**Table 1:** ^1^H-NMR (600 MHz) and ^13^C-NMR (150 MHz) spectra data of compounds **1** and **2** (in CD_3_OD, *J* in Hz, *δ* in ppm).

Position	1		2	
	*δ*_H_ (*J* in Hz)	*δ*_C_	*δ*_H_ (*J* in Hz)	*δ*_C_
2	4.81 (1H, s)	79.8	4.82 (1H, s)	79.8
3	4.26 (1H, m)	66.8	4.25 (1H, m)	66.7
4	2.85 (1H, dd, *J* = 17.2, 1.7)	29.8	2.85 (1H, dd, *J* = 17.2, 2.0)	29.7
	2.92 (1H, dd, *J* = 17.2, 4.5)		2.92 (1H, dd, *J* = 7.2, 4.4)	
5	–	157.4	–	157.4
6	6.20 (1H, s)	96.3	6.19 (1H, s)	96.3
7	–	151.6	–	151.7
8	–	106.2	–	106.2
9	–	153.6	–	153.5
10	–	105.4	–	105.3
1′	–	132.0	–	132.0
2′	7.06 (1H, d, *J* = 1.9)	115.1	7.03 (1H, d, *J* = 1.8)	115.1
3′	–	146.2	–	146.1
4′	–	145.9	–	145.8
5′	6.79 (1H, d, *J* = 8.1)	116.1	6.781 (1H, d, *J* = 8.2)	116.0
6′	6.84 (1H, dd, *J* = 8.1, 1.9)	119.1	6.83 (1H, dd, *J* = 8.2, 1.8)	119.1
1′′	–	135.2	–	135.8
2′′	6.43 (1H, s)	105.0	6.776 (1H, d, *J* = 2.0)	112.2
3′′	–	149.2	–	148.8
4′′	–	135.1	–	146.3
5′′	–	149.2	6.62 (1H, d, *J* = 8.2)	116.2
6′′	6.43 (1H, s)	105.0	6.50 (1H, dd, *J* = 8.2, 2.0)	119.4
7′′	4.60 (1H, br. d, *J* = 6.3)	36.2	4.60 (1H, br. d, *J* = 6.3)	35.7
8′′	2.91 (1H, dd, *J* = 15.9, 1.5)	37.5	2.90 (1H, dd, *J* = 15.9, 1.5)	37.8
	3.12 (1H, dd, *J* = 15.9, 7.5)		3.10 (1H, dd, *J* = 15.9, 7.4)	
9′′	–	171.0	–	170.9
3′′-OCH_3_	–	–	3.56 (3H, s)	56.0
3′′, 5′′-OCH_3_	3.59 (6H, s)	56.4	–	–

Compound **2** (Figure 1) was isolated as a pale yellow solid. The molecular formula of compound **2**, C_25_H_22_O_9_, was concluded from its HRESIMS, which showed quasi-molecular ion peaks at *m/z* 465.1189 [M−H]^−^ (calcd. for C_25_H_21_O_9_ 465.1186) and 467.1327 [M+H]^+^ (calcd. for C_25_H_23_O_9_ 467.1342) in the negative and positive modes, respectively. Its IR spectrum showed absorptions due to hydroxyl (3,453 cm^−1^) and carbonyl (1,745 cm^−1^) groups. UV absorptions at 202, 230 and 280 nm were observed. Detailed ^1^H, ^13^C, and HMBC spectra suggested **2** to be a phenylpropanoid-substituted epicatechin quite similar to compound **1**, but with differences in the substitution pattern of the E-ring. The ^1^H-NMR spectrum (Table 1) showed proton signals at *δ* 6.776 (1H, d, *J* = 2.0 Hz), *δ* 6.62 (1H, d, *J* = 8.2 Hz) and *δ* 6.50 (1H, dd, *J* = 8.2, 2.0 Hz), revealing the presence of a 1,3,4-trisubstituted aromatic ring. A methoxy group at *δ* 3.56 (3H, s) was observed. The HMBC spectrum showed correlation from the methoxy protons at *δ* 3.56 to *δ* 148.8 (C-3′′), indicating that the methoxy group was attached to C-3′′ in the E-ring. To define the absolute configuration of **2** at C-7′′, a circular dichroism investigation was undertaken. The CD spectrum showed a negative Cotton effects at 234 and 284 nm and a positive Cotton effect at 256 nm. Thus, C-7′′ also possessed the *R* configuration [13]. Accordingly, compound **2** was determined as epicatechin-(7,8-bc)-4*α*-(3-methoxy-4-hydroxy-phenyl)-dihydro-2(3*H*)-pyranone, and trivially named smiglabrone B.

Compound **3** (Figure 1) was obtained as a pale yellow solid. Its molecular formula was determined as C_18_H_18_O_6_ from the negative-ion HRESIMS with a quasi-molecular ion peak at *m/z* 329.1033 [M−H]^−^ (calcd. for C_18_H_17_O_6_ 329.1025). The IR spectrum of **3** showed characteristic absorption bands for hydroxyl (3,450 cm^−1^) and carbonyl (1,633 cm^-1^) groups. UV absorptions at 216 and 294 nm were observed. The ^1^H-NMR spectrum (Table 2) revealed a set of characteristic signals at *δ* 4.06 (1H, d, *J* = 11.0 Hz) and 3.96 (1H, d, *J* = 11.0 Hz) for one methylene linked to an oxygen atom, at *δ* 2.90 (1H, d, *J* = 14.5 Hz) and 2.86 (1H, d, *J* = 14.5 Hz) for one benzylic methylene, indicating that **3** possessed a homoisoflavanone skeleton. A trisubstitution of the A-ring was deduced from the occurence of a one aromatic proton singlet at *δ* 6.14, suggesting a substituent either at C-6 or at C-8. Four aromatic protons at *δ* 7.05 (2H, d, *J* = 8.5 Hz) and 6.70 (2H, d, *J* = 8.5 Hz) revealed a 1,4-disubstituted B-ring. In addition, an aromatic methoxyl group at *δ* 3.86 (3H, s) and a methyl group at *δ* 1.95 (3H, s) were also observed in the ^1^H-NMR spectrum. The ^13^C-NMR spectrum (Table 2) exhibited, in total, eighteen carbon resonances, including one aromatic carbon linked to a carbonyl at *δ* 101.8, one alkyl-substituted aromatic carbon at *δ* 126.8, one flavanone carbonyl carbon at *δ* 200.6, one methylene carbon having an oxygen function at *δ* 72.9, one benzylic methylene carbon at *δ* 40.8, one methoxyl carbon at *δ* 56.5, one methyl carbon at *δ* 7.0, and one quaternary carbon having an oxygen function at *δ* 73.6. The HMBC correlation of the methoxyl protons at *δ*3.86 with C-7 at *δ* 167.5 indicated that the linkage position of the methoxyl group was at C-7. The position for the methyl group attaching at C-6 was determined by correlation from methyl protons at *δ*1.95 to C-6 (*δ* 106.7). The long-range correlation from H-2 to C-9 (*δ* 162.7), C-4 (*δ* 200.6) and C-11 (*δ* 40.8), from H-11 to C-4 (*δ* 200.6) and C-2′ (*δ* 132.8) further confirmed the speculation of the structure of compound **3** (Figure 3). The planar structure of compound **3** was identified as 3,5-dihydroxy-7-methoxy-6-methyl-3-(4-hydroxybenzyl)chroman-4-one [14]. The absolute configuration of C-3 was assessed by optical its rotation (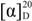 +178.72), indicating the *R* configuration at C-3 [15]. Consequently, the structure of compound **3** was identified as (3*R*)-3,5-dihydroxy-7-methoxy-6-methyl-3-(4-hydroxybenzyl)chroman-4-one, and trivially named smilachromanone.

**Table 2:** ^1^H-NMR (500 MHz) and ^13^C-NMR (125 MHz) spectra data of compound **3** (in CD_3_OD, *J* in Hz, *δ* in ppm).

Position	*δ*_H_ (*J* in Hz)	*δ*_C_
2	4.06 (1H, d, *J* = 11.0)	72.9
	3.96 (1H, d, *J* = 11.0)	–
3	–	73.6
4	–	200.6
5	–	161.6
6	–	106.7
7	–	167.5
8	6.14 (1H, s)	91.8
9	–	162.7
10	–	101.8
11	2.90 (1H, d, *J* = 14.5)	40.8
	2.86 (1H, d, *J* = 14.5)	–
1′	–	126.8
2′, 6′	7.05 (2H, d, *J* = 8.5)	132.8
3′, 5′	6.70 (2H, d, *J* = 8.5)	115.9
4′	–	157.5
7-OCH_3_	3.86 (3H, s)	56.5
6-CH_3_	1.95 (3H, s)	7.0

Compound **4** (Figure 1) was obtained as a brown-yellow powder. Its molecular formula was determined as C_14_H_12_O_4_ by negative-ion HRESIMS with quasi-molecular ion peaks at *m/z* 243.0659 [M-H]^−^ (calcd. for C_14_H_11_O_4_ 243.0657). The IR spectrum of **4** showed a characteristic hydroxyl absorption band (3,449 cm^−1^). UV absorptions at 220 and 306 nm were observed. The ^1^H-NMR spectrum (Table 3) exhibited proton signals at *δ* 6.48 (2H, d, *J* = 1.8 Hz) and 6.17 (1H, t, *J* = 1.8 Hz), suggesting the presence of a 1,3,5-trisubstituted aromatic ring in the molecule. The signals at *δ* 7.37 (1H, d, *J* = 16.8 Hz) and 6.96 (1H, d, *J* = 16.8 Hz) were attributed to a set of *trans*-olefinic protons. The remaining protons at *δ* 6.69 (1H, dd, *J* = 1.8, 7.8 Hz), 6.66 (1H, t, *J* = 7.8 Hz) and 7.02 (1H, dd, *J* = 1.8, 7.8 Hz) indicated the existence of a 1,2,3-trisubstituted aromatic ring. The ^13^C-NMR spectrum (Table 3) exhibited, in total, twelve carbon resonances including two olefinic carbons and ten aromatic carbons. The HMBC spectrum showed correlations from H-7 to C-1′ (*δ* 141.6) and C-2′, 6′ (*δ* 105.9), indicating that C-7, C-2′ and C-6′ were linked through C-1′; the correlations from H-8 to C-1 (*δ* 126.0), C-2 (*δ* 144.6) and C-6 (*δ* 118.3), suggesting that C-8, C-2 and C-6 were linked through C-1 (Figure 3). Accordingly, compound **4** was elucidated as (*E*)-3-(3,5-dihydroxystyryl)benzene-1,2-diol, and trivially named smiglastilbene.

**Table 3:** ^1^H-NMR (600 MHz) and ^13^C-NMR (150 MHz) spectra data of compound **4** (in CD_3_OD, *J* in Hz, *δ* in ppm).

Position	*δ*_H_ (*J* in Hz)	*δ*_C_
1	–	126.0
2	–	144.6
3	–	146.5
4	6.69 (1H, dd, *J* = 7.8, 1.8)	114.9
5	6.66 (1H, t, *J* = 7.8)	120.5
6	7.02 (1H, dd, *J* = 7.8, 1.8)	118.3
7	6.96 (1H, d, *J* = 16.8)	129.5
8	7.37 (1H, d, *J* = 16.8)	124.7
1′	–	141.6
2′, 6′	6.48 (2H, d, *J* = 1.8)	105.9
3′, 5′	–	159.7
4′	6.17 (1H, t, *J* = 1.8)	102.8

Compound **5** (Figure 1) was obtained as a white powder. Its molecular formula was determined as C_15_H_20_O_3_ by HREIMS with a molecular ion peak at *m/z* 248.1415 [M]^+^ (calcd. for C_15_H_20_O_3_ 248.1412). The IR spectrum of **5** showed characteristic absorption bands for hydroxyl (3,357 cm^−1^) and carbonyl (1,747 cm^−1^) groups. UV absorptions at 201 and 289 nm were observed. The ^1^H-NMR spectrum (Table 4) displayed four methyl groups at *δ* 0.76 (3H, d, *J* = 6.6 Hz), 0.98 (3H, d, *J* = 6.6 Hz), 2.30 (3H, s), 2.22 (3H, s), a proton signal at *δ* 5.72 (1H, d, *J* = 9.0 Hz) attached to the oxygenated carbon, and two methenyl proton signals at *δ* 2.75 (1H, m) and 1.76 (1H, m). In addition, a pair of vicinal coupled aromatic proton signals at *δ* 6.85 (1H, d, *J* = 8.4 Hz), 6.69 (1H, d, *J* = 8.4 Hz) indicated the presence of one 1,2,3,4-tetrasubstituted phenyl ring. The ^13^C-NMR spectrum (Table 4) exhibited one quaternary carbon signal attached to the O-atom at *δ* 179.6; four other quaternary carbons in the benzene ring at *δ* 156.2, 136.3, 128.9 and 125.2. Among these signals, the lower field carbon signal at *δ* 156.2 indicated that this C-atom was connected to the O-atom. The ^13^C-NMR spectrum also gave two tertiary carbons in the benzene ring at *δ* 130.7 and 116.2, one methylene carbon at *δ* 33.8, three methenyl carbons at *δ* 49.5, 85.1 and 31.3, four methyl carbons at *δ* 21.9, 19.9, 20.8 and 13.0. From the above information it was possible to deduce the presence of dihydrofuran in the molecule. The HMBC spectrum showed correlations from H-15 to C-5 (*δ* 136.3), C-9 (*δ* 156.2) and C-10 (*δ* 125.2), from H-14 to C-5 (*δ* 136.3), C-6 (*δ* 128.9) and C-7 (*δ* 130.7), thus indicating that two methyl groups are placed at C-10 and C-6, respectively. The HMBC correlations from H-12 and H-13 to C-11 (*δ* 31.3) and C-3 (*δ* 49.5) indicated that the presence of an isopropyl at C-3; the correlation from *δ* 5.72 (1H, d, *J* = 9.0 Hz, H-4) to C-5 (*δ* 136.3) suggested that the dihydrofuran was connected to C-5 in the benzene ring (Figure 3). The relative configuration of H-3 and H-4 was determined as *trans* by the 9.0 Hz coupling constant between H-3 and H-4. Irradiation of H-4 caused NOE enhancements of H-11, H-12 and H-13 in the 1D NOE spectra and indicated that isopropyl was positioned in the same plane with H-4. Thus, the NOE result further confirmed the *trans* conformation of H-4 and H-3. Consequently, compound **5** was elucidated as 5-(3-hydroxy-2,6-dimethylphenyl)-4-isopropyldihydrofuran-2(3*H*)-one, trivially named smiglactone.

**Table 4:** ^1^H-NMR (600 MHz) and ^13^C-NMR (150 MHz) spectra data of compound **5** (in CD_3_OD, *J* in Hz, *δ* in ppm).

Position	*δ*_H_ (*J* in Hz)	*δ*_C_
1	–	179.6
2	2.65 (2H, m)	33.8
3	2.75 (1H, m)	49.5
4	5.72 (1H, d, *J* = 9.0)	85.1
5	–	136.3
6	–	128.9
7	6.85 (1H, d, *J* = 8.4)	130.7
8	6.69 (1H, d, *J* = 8.4)	116.2
9	–	156.2
10	–	125.2
11	1.76 (1H, m)	31.3
12	0.76 (3H, d, *J* = 6.6)	21.9
13	0.98 (3H, d, *J* = 6.6)	19.9
14	2.30 (3H, s)	20.8
15	2.22 (3H, s)	13.0

Compound **6** (Figure 1) was obtained as a pale yellow solid. Its molecular formula was determined as C_15_H_16_O_5_ by negative-ion HRESIMS with quasi-molecular ion peaks at *m/z* 311.06917 [M+Cl]^−^, 338.08813 [M+NO_3_]^−^ and 587.16893 [2M+Cl]− (calcd. for C_15_H_16_O_5_Cl 311.06921, C_15_H_16_O_5_NO_3_ 338.08835 and C_30_H_32_O_10_Cl 587.16829, respectively). The IR spectrum of **6** showed a characteristic hydroxyl absorption band (3,336 cm^−1^). UV absorptions at 206, 225 and 278 nm was observed. The ^1^H-NMR spectrum (Table 5) showed proton signals at *δ* 7.30 (2H, d, *J* = 8.4 Hz) and 7.06 (2H, d, *J* = 8.4 Hz), revealing the presence of a 1,4-disubstituted aromatic ring, and the signals at *δ* 6.88 (1H, t, *J* = 2.4 Hz) and 6.97 (2H, d, *J* = 2.4 Hz) indicating the presence of a 1,3,5-trisubstituted aromatic ring. The ^1^H-NMR spectrum of **6** also indicated the presence of four hydroxyl groups at *δ* 11.24 (2H, br. s), 11.38 (1H, br. s) and 3.59 (1H, br. s), and one methoxy group at *δ* 3.28 (3H, s). The ^13^C-NMR spectrum (Table 5) exhibited, in total, eleven carbon resonances involving eight aromatic carbons, a methoxy carbon, and two oxygenated carbons. In the HMBC spectrum, the correlations from methoxy proton at *δ* 3.28 (3H, s) to C-8 (*δ* 89.5), and from H-8 to methoxy carbon at *δ* 56.8 suggested that the methoxy group was connected with C-8. The correlations from H-8 to C-10, 14 (*δ* 129.9), H-14 to C-10 (*δ* 129.9) indicated that C-8, C-10 and C-14 were linked through C-9; the correlations from H-7 to C-4, 6 (*δ* 107.1), H-6 to C-7 (*δ* 78.7) and H-4 to C-7 (*δ* 78.7) suggested that C-7, C-4 and C-6 were linked through C-5 (Figure 3). The large coupling constant between H-7 and H-8 (*J* = 7.8 Hz) suggested a *threo* conformation of C-7/C-8 [16,17]. Thus, the structure of compound **6** was determined as *threo*-5-[1-hydroxy-2-(4-hydroxyphenyl)-2-methoxyethyl] benzene-1,3-diol, trivially named smiglabrol.

**Table 5:** ^1^H-NMR (600 MHz) and ^13^C-NMR (150 MHz) spectra data of compound **6** (in C_5_D_5_N, *J* in Hz, *δ* in ppm).

Position	*δ*_H_ (*J* in Hz)	*δ*_C_
1, 3	–	159.7
2	6.88 (1H, t, *J* = 2.4)	102.9
4, 6	6.97 (2H, d, *J* = 2.4)	107.1
5	–	145.4
7	5.13 (1H, d, *J* = 7.8)	78.7
8	4.56 (1H, d, *J* = 7.8)	89.5
9	–	130.0
10, 14	7.30 (2H, d, *J* = 8.4)	129.9
11, 13	7.06 (2H, d, *J* = 8.4)	115.7
12	–	158.4
1, 3-OH	11.24 (2H, br. s)	–
7-OH	3.59 (1H, br. s)	–
8-OCH_3_	3.28 (3H, s)	56.7
12-OH	11.38 (1H, br. s)	–

### 2.2. Structural Elucidation of the Known Isolates

**Figure 4:**
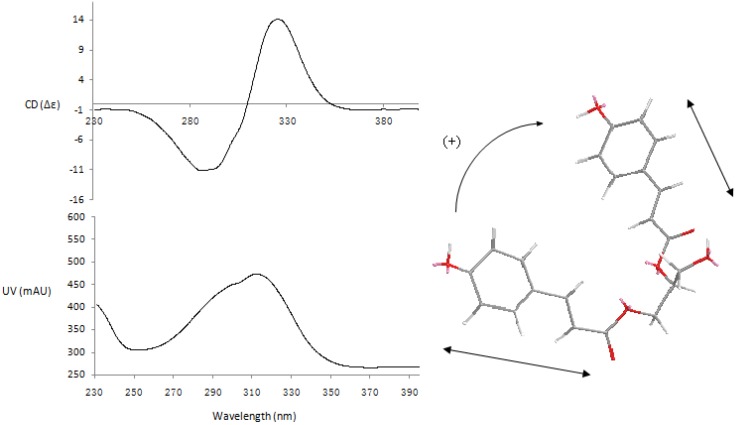
CD and UV spectra of compound **43**.

Compound **43** (Figure 2) was isolated as a pale yellow powder. Its molecular formula was determined as C_21_H_20_O_7_ from the negative-ion HRESIMS with a quasi-molecular ion peak at *m/z* 383.1133 [M−H]^−^ (calcd. for C_21_H_19_O_7_ 383.1131) and the positive negative-ion HRESIMS at *m/z* 407.1103 [M+Na]^+^ (calcd. for C_21_H_20_O_7_Na 407.1107). The IR spectrum of **43** showed characteristic absorption bands for hydroxyl (3,448 cm^−1^) and carbonyl (1,686 cm^−1^) groups. UV absorptions at 210, 228 and 310 nm were observed. The ^1^H-NMR spectrum exhibited signals of two *trans*-double bonds at *δ* 7.57 (1H, d, *J* = 16.2 Hz), 6.39 (1H, d, *J* = 16.2 Hz), 7.540 (1H, d, *J* = 15.6 Hz) and 6.38 (1H, d, *J* = 15.6 Hz). Eight aromatic protons at *δ* 7.541 (2H, d, *J* = 8.4 Hz), 6.77 (2H, d, *J* = 8.4 Hz), 7.53 (2H, d, *J* = 8.4Hz) and 6.76 (2H, d, *J* = 8.4 Hz), revealing the presence of two 1,4-disubstituted aromatic rings. Additionally, an acylated methine proton at *δ* 5.11 (1H, m), acylated methylene protons at *δ* 4.40 (1H, dd, *J* = 11.4, 3.0 Hz) and 4.25 (1H, dd, *J* = 12.0, 6.6 Hz), and hydroxymethyl protons at 3.60 (2H, t) suggested the presence of a 1,2-diacylglycerol moiety. The ^13^C-NMR spectrum showed two ester carbonyl carbon signals at *δ* 166.41 and 166.19, four olefinic carbons at *δ* 114.03, 145.21, 113.68 and 145.16, one oxymethine carbon at *δ* 72.13, and one oxymethylene carbon at *δ* 62.54. The HMBC correlation of H-1, H-6 with *δ* 166.19 (C-4), H-3′ with *δ* 166.41 (C-1′) further confirmed the presence of two non-equivalent *p*-coumarate moieties. The HMBC spectrum showed correlations from H-1, H-3 to *δ* 72.13 (C-2), H-1 to *δ* 59.67 (C-3), indicating that the hydroxymethyl and acylated methylene were linked through C-2. Thus, the planar structure of **43** was determined as 1,2-*O*-di-*trans*-*p*-coumaroylglycerol, that was reported as being isolated from stromata of *Epichloe typhina* on *Phleum pretense* [18]. To determine the absolute configuration of **43**, the exciton-coupled circular dichroism (ECCD) technique was applied [19,20]. The CD of **43** (Figure 4) exhibited a positive split between the two chromophores of the *p*-coumarate coupled with π→π^*^ transition (287 nm, Δε -11.12; 326 nm, Δε +14.09), indicating that the transition dipole moments of the two chromophores were oriented in a clockwise manner. This positive CD shows that the electric transition dipole of the *p*-coumarate chromophores constitute positive chirality. Thus the absolute configuration of the chiral center in **43** was deduced as 2*S*. Accordingly, compound **43** was elucidated as (2*E*, 2'*E*)-[(*S*)-3-hydroxypropane-1,2-diyl] bis[3-(4-hydroxyphenyl)acrylate], and the compound was trivially named smiglycerol.

Based on their 1D and 2D NMR, CD and MS spectroscopic data and comparison of the data with those reported in the literature, fifty-seven known compounds were identified. These included thirteen flavanones: taxifolin (**7**) [7], naringenin (**8**), dihydrokaempferol (**9**) [21], sakuranetin (**10**) [22], isoastilbin (**11**) [23], astilbin (**12**) [23], neoastilbin (**13**) [23], neoisoastilbin (**14**) [23], engeletin (**15**) [21], arthromerin B (**16**) [23,24], sinensin (**17**) [23,25], (2*R*, 3*R*)-taxifolin 3′-*O*-*β*-D-glucopyranoside (**18**), (2*S*,3*S*)-glucodistylin (**19**) [26], four flavanes: (-)-epicatechin (**20**) [13], (+)-catechin (**21**) [12], cinchonain Ib (**22**) [13], cinchonain Ia (**23**) [13], four flavonoids: apigenin (**24**), quercetin (**25**), luteolin (**26**), myricetin (**27**), one chalcone, kukulkanin B (**28**) [27], two aurones: 4,4′,6-trihydroxyaurone (**29**) [28], aureusidin (**30**) [29], six lignans: (-)-secoisolariciresinol (**31**) [30], 4-ketopinoresinol (**32**) [31], 1,4-bis(4-hydroxy-3,5-dimethoxyphenyl)-2,3-bis(hydroxymethyl)-1,4-butanediol (named smiglabranol by us for being utilized conveniently in future) (**33**) [32], (+)-lyoniresinol (**34**) [33], kompasinol A (**35**) [34], aiphanol (**36**) [35], three stilbenes: *trans*-resveratrol (**37**) [36], *trans*-piceid (**38**) [36], piceatannol (**39**) [36], six phenylpropanoids: *trans*-caffeic acid (**40**), 5-*O*-caffeoylshikimic acid (**41**) [21], 3-*O*-*p*-coumaroylshikimic acid (**42**) [37], (2*S*)-1,2-*O*-di-*trans*-*p*-coumaroylglycerol (named smiglycerol) (**43**) [18], juncusyl ester B (**44**) [38], 1-*O*-*p*-coumaroylglycerol (**45**) [39], nine phenolics: vanillin (**46**), *p*-hydroxy-benzaldehyde (**47**), acetovanillone (**48**), (+)-scytalone (**49**) [40], glucosyringic acid (**50**), protocatechuic acid (**51**), 3-methoxygallic acid (**52**), vanillic acid 1-*O*-*β*-D-glucopyranosyl ester (**53**), hydroxytyrosol (**54**), one triterpene, acetyl-11-keto-*β*-boswellic acid (**55**), three steroids: stigmasterol (**56**), *β*-sitosterol (**57**), daucosterol (**58**), and five other compounds: smilagenin (**59**), 5-hydroxymaltol (**60**), 5-hydroxyuridine (**61**), 2-methylbutanedioic acid-4-ethyl ester (**62**), isoselachoceric acid (**63**) (see Figure 2). Isolation of twenty-seven compounds (**10**, **17**, **26**–**36**, **42**, **43**, **45**, **46**, **48**–**50**, **52**–**55**, **60**–**62**) from the genus *Smilax* is reported here for the first time and fourteen compounds (**9**, **15**, **16**, **19**, **21**–**24**, **39**, **40**, **44**, **47**, **51**, **63**) were obtained from the rhizomes of *S. glabra* for the first time.

During our investigations, four phenylpropanoid-substituted epicatechins (compounds **1**, **2**, **22**, **23**), which were a class of flavan-3-ols substituted at the A ring with a C_6_-C_3_ unit, were obtained. To our knowledge, these have never been previously reported in *S. glabra.* Pharmacological studies revealed that compounds **22** and **23**possessed antioxidant [10], antifungal and antiviral [11] activities. This is the first report of the presence in *Smilax* of chalcone **28**, which was previously isolated from *Mimosa tenuefolia* [27]. Compounds with this skeleton were found to have antioxidant [41] and antitumor [42] activities. The two aurones, namely compounds **29** and **30**, were found to possess antiviral [28] and antioxidant [29] activities, respectively. Their isolation from the genus *Smilax* is reported here for the first time. Compound **36 **represents a stilbenolignan skeleton in which a stilbene moiety is linked with a phenylpropane unit through a dioxane bridge. It was previously reported from *Aiphanes aculeate* and exhibited significant inhibitory activities against cyclooxygenases-1 and -2 [35]. Compound **35** is also a stilbenolignan, which was isolated from *Koompassia malaccensis* [34] and *Syagrus romanzoffiana* and had anti-*α*-glucosidase activity [43]. This is first report of the presence of compounds with this type of skeleton in *Smilax* plants.

On the basis of previous phytochemical studies and our investigations, flavonoids are the main constituents in the rhizomes of *S. glabra*, and especially flavanones were relatively more abundant than other kinds of flavonoids. It was reported that the contents of the five flavonoids were in the range of 0.0290–1.06, 0.0128–0.0543, 1.53–11.3, 0.449–13.7 and 0.552–4.837 mg/g crude drugs for taxifolin, neoastilbin, astilbin, neoisoastilbin and isoastilbin, respectively [8]. In addition, quantitative analysis showed that the content of engeletin was in the range of 0.14–3.1 mg/g crude drug [44]. Previous phytochemical investigations showed that fourteen flavonoids were isolated the rhizomes of *S. glabra*, which included ten flavanones: taxifolin, astilbin, neoastilbin, isoastilbin, neoisoastilbin, (2*R*,3*R*)-taxifolin-3′-*O*-*β*-D-glucopyranoside, isoengelitin, naringenin, engeletin, smitilbin [45,46,47], two flavonoids: quercetin, quercetin 4′-*O*-*β*-D-glucoside [48], one isoflavone, 7,6′-dihydroxy-3'-methoxy-isoflavone [46], and one flavane, (-)-epicatechin [49]. 

### 2.3. Antimicrobial Activity

The MIC obtained in the antimicrobial assessment of *S. glabra* extracts demonstrated considerable activity in the ethanolic extract, ethyl acetate fraction and *n*-butanol fraction against *S. aureus* ATCC6538 (50 μg/mL). The ethyl acetate fraction and water fraction showed activity against *C. albicans* SC5314 and *S. aureus* ATCC6538 with MIC values of 200 μg/mL. This is the first report on the antifungal properties of *S. glabra*. 

Thirty compounds were tested for their antimicrobial activity (Table 6). The results demonstrated that seventeen of these compounds were found to have antibacterial activity against Gram-positive bacteria and ten compounds displayed activity against the tested fungus, while eight compounds were found to be antibacterial against Gram-negative bacteria. As for the four flavan-3-ols (Figure S1, Table S1), **1**, **20** and **22** demonstrated antimicrobial properties, whereas **21** was inactive in the concentration range tested. Compound **1** exhibited activity against all tested microorganisms, which was stronger against *C. albicans* SC5314 with a MIC value of 0.146 mM and weaker against the Gram-positive and Gram-negative bacteria where the MIC values were in the range of 0.302-0.604 mM. Compound **22 **showed activity against methicillin-resistant *S. aureus* and *S. aureus* ATCC6538 with a MIC value of 0.0801 mM and activity against *C. albicans* SC5314 and *E. faecalis* with MIC valued of 0.160 mM. Compound **20** was only found to be active against methicillin-resistant *S. aureus*, *S. aureus* ATCC6538 and *E. faecalis*. Compounds **1**, **20** and **22** all possessed epicatechin units, while **21** was identified as catechin. The result suggested that the antimicrobial activity was influenced by the stereochemistry at C2 and C3.

A similar situation happens with respect to some of the investigated flavanones. Compound **7** was inactive and its flavanonol rhamnoside isomers (compounds **12**, **13** and **14**) showed antimicrobial activity except for **11** (Figure S2, Table S2). Among them, compound **14**, with 2*S*,3*R* configuration, presented the best activity and showed inhibitory effects toward all of the tested microorganisms. This indicates that the stereochemistry at C2 and C3 of taxifolin largely governs the potency of inhibition, and controls the steric configuration of the rhamnose moiety. Taxifolin (**7**), astilbin (**12**), neoastilbin (**13**) and isoastilbin (**11**) have been found to display antimicrobial activity against *Streptococcus sobrinus* with MIC values in the 0.500–0.740 mM range. Among these compounds, neoastilbin showed an especially potent GTase inhibitory activity, which suggested that the antimicrobial activity was related to stereochemistry [7]. To some extent, our results mirror the previous study [7].

Compound **9** and its glycoside (compound **17**) demonstrated activity against methicillin-resistant *S. aureus* and *S. aureus* ATCC6538, whereas **15** and **16** did not show inhibitory effects toward these microorganisms. This implied that for the C ring, hydroxylation at position 3 improved the activity of flavanones (Figure S3, Table S3). Compound **10** was found to be antibacterial against methicillin-resistant *S. aureus*, *S. aureus* ATCC6538 and *E. faecalis* with MIC values of 0.524, 0.524 and 1.05 mM. The homoisoflavanone **3** showed antibacterial activity against *S. aureus* ATCC6538, with a MIC value of 0.303 mM, and weaker activity against *C. albicans* SC5314, methicillin-resistant *S. aureus* and *E. faecalis* with a uniform MIC of 0.605 mM.

All the investigated stibenes (compounds **4**, **37**–**39**) displayed antimicrobial activity against the microorganisms tested (Figure S4, Figure S5, Table S4). Compound **37** showed antibacterial activity against methicillin-resistant *S. aureus*, *S. aureus* ATCC6538 and *E. faecalis* with MIC values of 0.159, 0.0794 and 0.159 mM, respectively. It appeared that compound **38** which was the glycosylated version of resveratrol (**37**) had much weaker activity than its parent, which was reported to have antibacterial activity against *Streptococcus mutans* and *Streptococcus sanguis* with MIC values of 0.219 and 0.110 mM, respectively, but piceid (**38**) did not inhibit microbial growth [50].The structures of compound **4** and **39 **were quite similar, except for the position of the hydroxyl, while the activity of **4** was better than **39**. They displayed inhibitory activity toward *S. aureus* ATCC6538 and methicillin-resistant *S. aureus* with MIC values of 0.205 and 0.409 mM, respectively. We infer that the hydroxyl position may influence antimicrobial effect, and the activity of 2,3-hydroxyl-substituted compounds was better than that of 3,4-hydroxyl-substituted compounds. The stibenolignan **36** showed antimicrobial activity against all the microorganisms tested. We suppose that the presence of a stilbene moiety in compound **36**contributes to the antimicrobial effect.

**Table 6:** Minimum inhibitory concentrations (MICs, mM) of the selected compounds obtained from the rhizomes of *S. glabra.*

Compound	EC	PA	KP	MRSA	SA	EF	CA
**1**	0.604	0.604	0.604	0.302	0.302	0.302	0.146
**3**	>1.21	>1.21	>1.21	0.605	0.303	0.605	0.605
**4**	1.64	1.64	1.64	0.409	0.205	0.819	0.819
**9**	>2.10	>2.10	>2.10	2.10	2.10	>2.10	>2.10
**10**	>2.10	>2.10	>2.10	0.524	0.524	1.05	>2.10
**11**	>1.33	>1.33	>1.33	>1.33	>1.33	>1.33	>1.33
**12**	>1.33	>1.33	>1.33	>1.33	>1.33	>1.33	0.666
**13**	>1.33	>1.33	>1.33	1.33	>1.33	>1.33	>1.33
**14**	1.33	1.33	1.33	1.33	0.666	1.33	1.33
**15**	>1.38	>1.38	>1.38	>1.38	>1.38	>1.38	>1.38
**16**	>1.33	>1.33	>1.33	>1.33	>1.33	>1.33	>1.33
**17**	>1.33	>1.33	>1.33	1.33	1.33	1.33	>1.33
**20**	>2.07	>2.07	>2.07	1.03	0.517	1.03	>2.07
**22**	>1.33	0.663	>1.33	0.0801	0.0801	0.160	0.160
**33**	>0.880	>0.880	>0.880	>0.880	>0.880	>0.880	>0.880
**36**	0.663	0.663	0.663	1.33	0.663	1.33	0.332
**37**	>2.63	>2.63	>2.63	0.159	0.0794	0.159	0.657
**38**	>1.54	>1.54	>1.54	0.768	0.768	1.54	>1.54
**39**	1.64	1.64	1.64	0.409	0.205	>1.64	0.819
**44**	2.52	2.52	2.52	0.630	0.630	1.26	0.630
**49**	3.09	3.09	3.09	3.09	1.55	3.09	3.09
**51**	>2.60	>2.60	>2.60	>2.60	>2.60	>2.60	>2.60
Cipro	0.00302	0.00302	0.00604				
Van				0.000690	0.000690	0.00138	
Keto							0.0000301

EC, *Escherichia coli*; PA, *Pseudomonas aeruginosa* PA01; KP, *Kiebsiella pneumonia* (clinical isolate); MRSA, methicillin-resistant *Staphylococcus aureus* (clinical isolate); SA, *Staphylococcus aureus* ATCC6538; EF, *Enterococcus faecalis*; CA, *Canidia albicans* SC5314; Cipro, ciprofloxacin; Van, vancomycin; Keto, ketoconazole.

## 3. Experimental

### 3.1. General

Optical rotations were measured with a Rudolph Research Analytical Autopol IV Automatic Polarimeter. IR spectra were recorded on a Thermo Nicolet Nexus 470 FT-IR spectrophotometer with KBr pellets. UV spectra were recorded on a Varian Cary Eclipse 300 spectrometer. 1D and 2D NMR experiments were performed on a Varian INOVA 500 or Bruker DRX 400 or 600 spectrometers with tetramethylsilane (TMS) as internal reference, and chemical shifts were expressed in *δ* (ppm). HR-ESI-MS and HR-EI-MS data were obtained on a Shimadzu LCMS-IT-TOF mass spectrometer and a Bruker Daltonics APEXII FT-ICR mass spectrometer, respectively. Column chromatography was performed on silica gel (100–200 mesh, 200–300 mesh, Qingdao Marine Chemical Inc., Qingdao, China), Sephadex LH-20 (Amersham Pharmacia Biotech Ltd., Beijing, China), Toyopearl HW-40 (Amersham Pharmacia Biotech Ltd., Beijing, China), macroporous resin D101 (Cangzhou Baoen Adsorptive Material Technology Co., Ltd., Hebei, China) and RP-C_18_ (Daiso, Osaka, Japan). TLC was performed with glass precoated silica gel GF_245_ plates (Qingdao Marine Chemical Inc., Qingdao, China). MPLC was performed using a Lisui EZ Purify III System including pump manager P03, detector modules P02, and fraction collector P01 (Shanghai Li Sui Chemical Engineering Co., Ltd., Shanghai, China) and columns packed with RP-18 silica gel (Merck, Darmstadt, Germany). Semi-preparative high performance liquid chromatography was carried out on an Agilent 1200 system equipped with a diode array detector working in the range of 190–500 nm. Samples were separated on a Zorbax SB-C_18_ (250 mm × 9.4 mm, 5 μm, Agilent) column. 

### 3.2. Plant Material

Two batches of the dried sliced rhizomes of *Smilax glabra* Roxb. were purchased from Beijing Ben Cao Fang Yuan Pharmaceutical Co. Ltd. (Beijing, China) in May 2009 and December 2011, respectively, and were identified by Prof. Shao-Qing Cai (State Key Laboratory of Natural and Biomimetic Drugs, School of Pharmaceutical Sciences, Peking University, Beijing, China). The voucher specimens (No.6176 and No.6836) were deposited in the Herbarium of Pharmacognosy, School of Pharmaceutical Sciences, Peking University (Beijing, China).

### 3.3. Extraction and Isolation

The air-dried and powdered rhizomes of *S. glabra* (20 kg, No.6176) were extracted three times with 95% ethanol (120 L, 100 L, 100 L) and 50% ethanol (100 L × 3) under reflux, respectively. After evaporation of the combined extracts, the residue (2,552 g) was suspended in 10 L of water, and then partitioned successively with petroleum ether (10 L × 4), ethyl acetate (EtOAc, 10 L × 4), and *n*-butanol (*n*-BuOH, 10 L × 4) to afford the corresponding petroleum-ether-soluble, EtOAc-soluble, and *n*-BuOH-soluble fractions after concentration of the solutiond under vacuum. The ethyl acetate extract (140 g) was subjected to silica gel (200-300 mesh, 2300 g) column chromatography and eluted with a gradient of CHCl_3_-MeOH (20:1–0:1, v/v) to give 13 fractions TE1-TE13. Fraction TE2 (6.5 g) was applied to a silica gel (200–300 mesh, 150 g) column using a gradient of petroleum ether-acetone (80:1–1:4, v/v) to get the corresponding subfractions. Compounds **63** (3.5 mg), **57 **(328 mg), **56** (16.1 mg), **5** (4.5 mg), **48** (1.1 mg), **55** (4.4 mg) and **8** (30.8 mg) were obtained from TE2 by silica gel column chromatography combined with semipreparative HPLC (CH_3_CN-H_2_O gradient elution). Fraction TE3 (9.5 g) was subjected to a silica gel (200–300 mesh, 300 g) column eluting with a gradient of petroleum ether-acetone (40:1-1:4, v/v) to give nine subfractions (E3-1-E3-9). E3-4 and E3-6 were then separated by semipreparative HPLC (CH_3_CN-H_2_O gradient elution) to afford **46** (4.0 mg), **3 **(10.2 mg) and **32 **(5.3 mg). Subfraction E3-8 was subjected to a Sephadex LH-20 column (2.5 × 57 cm) eluting with CHCl_3_-MeOH (1:1, v/v) and was further purified by semipreparative HPLC to yield **10** (5 mg). Fraction TE4 (7.5 g) was chromatographed on a silica gel (200-300 mesh, 230 g) column using a gradient of petroleum ether-acetone (6:1–1:4, v/v) to get subfractions. Compounds **49** (2.4 mg), **31 **(3.9 mg) and **9 **(19.3 mg) were isolated from TE4 using MPLC (ODS column) and semipreparative HPLC (CH_3_CN-H_2_O gradient elution). Compound **38 **(8.1 mg) was obtained by recrystallization. Compound **37 **(2.3123 g) was obtained from fraction TE5 (6.6 g) using recrystallization, while the remaining portion was subjected to a silica gel (200-300 mesh, 190 g) column eluting with a gradient of CHCl_3_-MeOH (30:1-1:2, v/v) to give 12 subfractions (E5-1-E5-12). Compounds **6 **(2.3 mg), **33** (13.8 mg) and **34** (1.9 mg) were obtained from TE5 through Sephadex LH-20 column chromatography and reverse-phase semipreparative HPLC. Compound **58** (106.4 mg) was obtained from fraction TE6 (5.7 g). The remaining portion of TE6 was subjected to a silica gel (200–300 mesh, 170 g) column eluting with a gradient of CHCl_3_-MeOH (40:1-0:1, v/v) and then purified by semipreparative HPLC to yield **24** (12.7 mg). Fraction TE7 (6.7 g) was separated on a silica gel (200-300 mesh, 260 g) column eluting with a gradient of cyclohexane-acetone (3:1-0:1, v/v) to obtain 11 subfractions (E7-1-E7-11). Compounds **7 **(22.3 mg), **21** (25.4 mg) and **59** (3.1 mg) were obtained from TE7 by repeated Sephadex LH-20 chromatography and MPLC. Fraction TE8 (10.7 g) was subjected to a silica gel (200-300 mesh, 270 g) column eluting with a gradient of CHCl_3_-MeOH (10:1-0:1, v/v) to give eight subfractions (E8-1-E8-8). Subfraction E8-6 was separated on a silica gel (200–300 mesh, 50 g) column and eluted with a gradient of CHCl_3_-MeOH-H_2_O (7:1:0.1-1:1:0.1, v/v/v) to afford **20 **(36.6 mg). Compound **11 **(2.0265 g) was obtained from fraction TE9 (35 g), while the remaining portion was applied to a silica gel (200–300 mesh, 700 g) column eluting with a gradient of CHCl_3_-MeOH (6:1-1:2, v/v) to yield **12 **(9.1454 g). The obtained subfractions were separated by repeated Sephadex LH-20, Toyopearl HW-40 column chromatography and further separated by semipreparative HPLC to yield **13** (85.1 mg), **14** (18.3 mg), **15** (19.7 mg) and **16** (7.6 mg). Fraction TE11 (10.1 g) was subjected to a silica gel (200–300 mesh, 290 g) column eluting with a gradient of cyclohexane-acetone (2:1-0:1, v/v) and further separated by semipreparative HPLC to yield **47** (15.8 mg). 

The *n*-butanol extract (183.9 g) was dissolved in water and passed through a column of D101 macroporous adsorptive resin (4 L) with water, 10%, 20%, 30%, 40%, 60%, 80%, 95% EtOH as eluents, respectively, to afford eight fractions TB1-TB8. Fraction TB2 was separated using MPLC (ODS column, CH_3_CN-H_2_O gradient elution). The obtained subfractions were submitted to Sephadex LH-20 column chromatography and further separated by semipreparative HPLC (CH_3_CN-0.1%TFA gradient elution) to yield **50** (104.6 mg), **61** (8.4 mg) and **53** (8.3 mg). Fraction TB4 was subjected to Sephadex LH-20, Toyopearl HW-40 column repeatedly and was further separated by semipreparative HPLC to yield **19** (3 mg), **41** (9 mg), **18** (3 mg) and **17** (6.4 mg).

The second batch of crude drug, air-dried and powdered rhizomes of *S. glabra* (42 kg, No. 6836) were extracted and isolated. The extraction method was the same as for the first batch. The ethyl acetate extract (282 g) was applied to a silica gel (100–200 mesh, 4970 g) column and eluted with a gradient of CHCl_3_-MeOH (30:1-0:1, v/v) to give 17 fractions GE1-GE17. Fraction GE5 (8 g) was separated on Sephadex LH-20 column (2.7 × 62 cm) repeatedly with CHCl_3_-MeOH (1:1, v/v) as eluent to give the corresponding subfractions, which were repeatedly subjected to Sephadex LH-20 column chromatography and further purified by semipreparative HPLC (CH_3_CN-0.03%TFA gradient elution) to afford **51** (30.2 mg), **40** (28.4 mg), **29** (6 mg), **52** (6 mg), **28** (3.1 mg) and **36** (4.5 mg). Fraction GE6 (7.1 g) was sunjected to repeated Sephadex LH-20, Toyopearl HW-40 column chromatography and further separated via semipreparative HPLC to get **54** (6.5 mg), **60** (1.9 mg) and **43** (2 mg). Fraction GE8 (3 g) and GE9 (4.5 g) were treated by the similar method as GE6 to yield **25** (14.5 mg), **26** (3.1 mg), **44** (6 mg), **45** (4 mg), **35** (5.7 mg), **4** (21.1 mg) and **30** (5 mg). Fraction GE10 (5 g) was repeatedly submitted to Sephadex LH-20 column chromatography and further purified by semipreparative HPLC to afford **42** (1.7 mg), **1** (4.4 mg), **2 **(2.5 mg), **39** (24.5 mg) and **27** (2 mg), while GE12 (5 g) was separated using the similar method as GE10 to yield **22** (8.3 mg) and **23** (2.8 mg).

### 3.4. Spectroscopic Data

*Smiglabrone A* (**1**). Pale yellow solid; mp 175–177 °C; 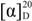 63.6 (*c* 0.44, MeOH); CD (2.72 × 10^−4^ MeOH): Δε (nm) 0.49 (214), −14.46 (235), 0.41 (259), −4.77 (284); UV λ_max_ (MeOH): 202, 230, 280 nm; IR (KBr) *ν*_max_ (cm^−1^): 3,452, 2,920, 2,850, 1,751, 1,676, 1,620, 1,520, 1,466, 1,368, 1,278, 1,202, 1,161, 1,112, 1,064, 997, 834, 724; HR-ESI-MS *m/z*: 495.1299 [M−H]^−^ (calcd. for C_26_H_23_O_10_ 495.1291); ^1^H- and ^13^C-NMR spectroscopic data see Table 1.

*Smiglabrone B* (**2**). Pale yellow solid; mp 172–174 °C; 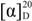 −80.0 (*c* 0.20, MeOH); CD (4.60 × 10^−4^ MeOH): Δε (nm) 2.02 (214),−18.04 (234),1.29 (256),−4.84 (284); UV λ_max_ (MeOH): 202, 230, 280 nm; IR (KBr) *ν*_max_ (cm^−1^): 3,453, 2,921, 2,853, 1,745, 1,629, 1,542, 1,458, 1,261, 1,165, 1,112, 894, 802; HR-ESI-MS *m/z*: 465.1189 [M−H]^−^ (calcd. for C_25_H_21_O_9_ 465.1186), 467.1327 [M+H]^+^ (calcd. for C_25_H_23_O_9_ 467.1342); ^1^H- and ^13^C-NMR spectroscopic data see Table 1.

*Smilachromanone* (**3**). Pale yellow solid; mp 81–83 °C; 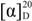 +178.72 (*c* 0.47, MeOH); CD (1.94 × 10^−4^ MeOH): Δε (nm) +13.43 (260), +6.22 (312), −29.23 (290); UV λ_max_ (MeOH): 216, 294 nm; IR (KBr) *ν*_max_ (cm^−1^): 3,450, 2,921, 1,633, 1,436, 1,376, 1,314, 1,243, 1,165, 1,058, 897, 795; HR-ESI-MS *m/z*: 329.1033 [M-H]^−^ (calcd. for C_18_H_17_O_6_ 329.1025); ^1^H- and ^13^C-NMR spectroscopic data see Table 2.

*Smiglastilbene *(**4**). Brown-yellow powder; mp 179–181 °C; UV λ_max_ (MeOH): 220, 306 nm; IR (KBr) *ν*_max_ (cm^−1^): 3,449, 1,674, 1,595, 1,475, 1,382, 1,346, 1,275, 1,201, 1,160, 996, 832; HR-ESI-MS *m/z*: 243.0659 [M-H]^−^ (calcd. for C_14_H_11_O_4_ 243.0657); ^1^H- and ^13^C-NMR spectroscopic data see Table 3.

*Smiglactone* (**5**). White powder; mp 88–90 °C; 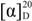 +120 (*c* 0.1, MeOH); CD (8.06 × 10^−4^ MeOH): Δε (nm) +34.11 (214)，-10.21 (298); UV λ_max_ (MeOH): 201, 289 nm; IR (KBr) *ν*_max_ (cm^−1^): 3,357, 1,747, 1,592, 1,489, 1,466, 1,373, 1,321, 1,279, 1,219, 1,160, 1,081, 1,049, 983, 817, 702; HR-EI-MS *m/z*: 248.1415 [M]^+^ (calcd. for C_15_H_20_O_3_ 248.1412); ^1^H- and ^13^C-NMR spectroscopic data see Table 4.

*Smiglabrol* (**6**). Pale yellow solid; mp 128–130 °C; 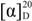 −156.5(*c* 0.08, MeOH); UV λ_max_ (MeOH): 206, 225, 278nm; IR (KBr) *ν*_max_ (cm^−1^): 3,360, 2,927, 1,678, 1,611, 1,515, 1,455, 1,378, 1,341, 1,303, 1,245, 1,158, 1,092, 1,058, 1,007, 923, 838, 718, 695; HR-ESI-MS *m/z*: 311.06917 [M+Cl]^−^, 338.08813 [M+NO_3_]^−^, 587.16893 [2M+Cl]^−^ (calcd. for C_15_H_16_O_5_Cl 311.06921, C_15_H_16_O_5_NO_3_ 338.08835, C_30_H_32_O_10_Cl 587.16829, respectively); ^1^H- and ^13^C-NMR spectroscopic data see Table 5.

### 3.5. Antimicrobial Assay

The screening for *in vitro* antibacterial activity was performed according to the Antimicrobial Susceptibility Testing Standards outlined by the Clinical and Laboratory Standards Institute (CLSI, formerly NCCLS) [51]. The microorganisms used were: *E. coli*, *P. aeruginosa* PA01, *K. pneumonia*, methicillin-resistant *S. aureus* and *S. aureus* ATCC6538, *Enterococcus faecalis*. Vancomycin was used as positive control drug for methicillin-resistant *S. aureus*, *S. aureus* ATCC6538 and *E. faecalis* assay, and ciprofloxacin for *P. aeruginosa* PA01, *E. coli* and *K. pneumonia* assay. MIC (minimal inhibition concentration) here is defined as the lowest concentration of compound that results in inhibition of visible bacterial growth (no turbidity) compared with the positive control antibiotics. All tests were performed in triplicate. *Canidia albicans* SC5314 was used as a test strain for antifungal bioassay. The experiments were carried out using a broth microdilution protocol modified from the Clinical and Laboratory Standards Institute M-27A methods [52]. The antifungal positive control was ketoconazole and antifungal MICs were determined by measuring and comparing the optical diversities of the blank control and tested wells. All tests were performed in triplicate.

## 4. Conclusions

In this study, six new phenolic compounds **1**–**6**, together with fifty-seven known compounds **7**–**63**, were isolated and identified from the rhizomes of *S. glabra*. Twenty-seven of these compounds **10**, **17**, **26**–**36**, **42**, **43**, **45**, **46**, **48**–**50**, **52**–**55**, **60**–**62** have never been reported from the genus *Smilax* before. In addition, this is the first report of the presence of the homoisoflavanone **3**, chalcone **28**, aurones **29**, **30** and stibenolignans **35**, **36** in this genus. The antimicrobial results revealed the effective constituents of this crude drug for clinical use. The structure types, stereo configuration and substituent groups appeared to be important structural factors that determined their antimicrobial properties. The stereochemistry at C2 and C3 of flavan-3-ols and flavanones largely govern the potency of antimicrobial activity. In addition, hydroxylation at position 3 of the C ring is essential for the activity of flavanones. It appeared that the glycosylation at position 3 of resveratrol resulted in the remarkable decrease of the antimicrobial effect. Our research revealed that the three stilbenes (**4**, **37**, **39**), two flavan-3-ols (**1**, **22**), one homoisoflavanone (**3**), one phenylpropanoid (**44**) and one stilbenolignan (**36**) present in the crude extract displayed better antimicrobial activity than the other investigated compounds. The lack of potency in some of the compounds isolated in this study appears to reinforce the view that herbal drug extracts may be superior to single constituents due to synergistic effects. Further studies are warranted to reveal the mechanisms of the active compounds found in *S. glabra* rhizomes underlying their antimicrobial properties.

## Supplementary Materials

Supplementary materials can be accessed at: http://www.mdpi.com/1420-3049/18/5/52658/s1.

## References

[1] Wang F.Z., Tang J, Chen X.Q., Zhang Z.Y., Dai L.K., Liang S.J., Tang Y.C., Liu L.,  Lang K.Y. (1978). Flora Reipublicae Popularis Sinicae.

[2] Song L., Wu Y., Hu L., Zhang G., Xu G., Xiao P., Ling Y., Ding X., Cao C., Li Y. (1999). Zhong Hua Ben Cao.

[3] Pharmacopoeia Commission of PRC (2010). Pharmacopoeia of the People’s Republic of China.

[4] Gao Y.J., Su Y.H., Qu L.K., Xu S., Meng L., Cai S.Q., Shou C.C. (2011). Mitochondrial apoptosis contributes to the anti-cancer effect of *Smilax glabra* Roxb. Toxicol. Lett..

[5] Zhang Q.F., Zhang Z.R., Cheung H.Y. (2009). Antioxidant activity of *Rhizoma Smilacis Glabrae* extracts and its key constituent-astilbin. Food Chem..

[6] Ji L.L., Fan Y.M. (2002). Antibacterial activity of extracts from *Smilax glabra*. Life Sci. Res..

[7] Kuspradini H., Mitsunaga T., Ohashi H. (2009). Antimicrobial activity against *Streptococcus sobrinus* and glucosyltransferase inhibitory activity of taxifolin and some flavanonol rhamnosides from kempas (*Koompassia malaccensis*) extracts. J. Wood Sci..

[8] Chen L., Yin Y., Yi H.W., Xu Q., Chen T. (2007). Simultaneous quantification of five major bioactive flavonoids in *Rhizoma Smilacis Glabrae* by high-performance liquid chromatography. J. Pharmaceut. Biomed..

[9] You J.G. (2002). Summary on clinical application of the rhizomes of *Smilax glabra* Roxb. Fujian J. Tradit. Chin. Med..

[10] Ao C.W., Higa T., Khanh T.D., Upadhyay A., Tawata S. (2011). Antioxidant phenolic compounds from *Smilax sebeana* Miq. Food Sci. Technol..

[11] Ming D.S., López A., Hillhouse B.J., French C.J., Hudson J.B., Towers G.H.N. (2002). Bioactive constituents from *Iryanthera megistophylla*. J. Nat. Prod..

[12] Foo L.Y. (1987). Phenylpropanoid derivatives of catechin, epicatechin and phylloflavan from *Phyllocladus trichomanoides*. Phytochemistry.

[13] Nonaka G.I., Nishioka I. (1982). Tannins and related compounds VII phenylpropanoid-substituted epicatechin, cinchonains from *Cinchona succirubra*. Chem. Pharm. Bull..

[14] Watanabe Y., Sanada S., Ida Y., Shoji J. (1985). Comparative studies on the constituents of Ophiopogonis tuber and its congenes. IV. Studies on the homoisoflavonoids of the subterranean part of *Ophiopogon ohwii* OKUYAMA and *O. jaburan* (KUNTH) LODD. Chem. Pharm. Bull..

[15] Tang Y.P., Yu B., Hu J., Wu T., Hui H.Z. (2002). Three new homoisoflavanone glycosides from the bulbs of *Ornithogalum caudatum*. J. Nat. Prod..

[16] Spassov S.L. (1969). Nuclear magnetic resonance spectra, configuration and conformation of diastereomers: 3-substituted 2,3-diphenylpropanoic acids and their methyl esters. Tetrahedron.

[17] Yuan Z., Li X. (2003). NMR methods for determining the configuration of 8-*O*-4′ neolignans. Chin. J. Mag. Reson..

[18] Koshino H., Terada S., Yoshihara T., Sakamura S., Shimanuki T., Sato T., Tajimi A. (1988). Three phenolic acid derivatives from stromata of *Epichloe typhina* on *Phleum pretense*. Phytochemistry.

[19] Berova N., Nakanishi K. (2000). Circular Dichroism: Principles and Applications.

[20] Adam W., Humpf H.U., Korb M.N., Schreier P. (1997). The configurational assignment of the optically active 5-(1-hydro-peroxyethyl)-3-ethoxycarbonyl-2-methylfuran and its alcohol by exciton-coupled circular dichroism (ECCD). Tetrahedron Asymmetr..

[21] Wungsintaweekul B., Umehara K., Miyase T., Noguchi H. (2011). Estrogenic and anti-estrogenic compounds from the Thai medicinal plant, *Smilax corbularia* (Smilacaceae). Phytochemistry.

[22] Zhang X.F., Hung T.M., Phuong P.T., Ngoc T.M., Min B.S., Song K.S., Seong Y.H., Bae K. (2006). Anti-inflammatory activity of flavonoids from *Populus davidiana*. Arch. Pharm. Res..

[23] Kasai R., Hirono S., Chou W.H., Tanaka O., Chen F.H. (1988). Sweet dihydroflavonol rhamnoside from leaves of *Engelhardtia chrysolepis*, a Chinese folk medicine, Hung-qi. Chem. Pharm. Bull..

[24] Kang J, Xie C.H., Li Z.M., Nagarajan S, Schauss A.G., Wu T., Wu X.L. (2011). Flavonoids from acai (*Euterpe oleracea* Mart.) pulp and their antioxidant and anti-inflammatory activities. Food Chem..

[25] Si C.L., Wu L., Zhu Z.Y. (2009). Phenolic glycosides from *Populus davidiana* bark. Biochem. Syst. Ecol..

[26] Dübeler A., Voltmer G., Gora V., Lunderstädt J., Zeeck A. (1997). Phenols from *Fagus sylvatica* and their role in defense against *Cryptococcus fagisuga*. Phytochemistry.

[27] Dominguez X.A., Garcia S., Williams H.J., Ortiz C., Scott A.N., Beibenspies J.H.  (1989). Kukulkanins A and B, new chalcones from *Mimosa tenuefolia*. J. Nat. Prod..

[28] Haudecoeur R., Ahmed-Belkacem A., Yi W., Fortune A., Brillet R., Belle C., Nicolle E., Pallier C., Pawlotsky J.M., Boumendjel A. (2011). Discovery of naturally occur ring aurones that are potent allosteric inhibitors of hepatitis C virus RNA-dependent RNA polymerase. J. Med. Chem..

[29] Detsi A., Majdalani M., Kontogiorgis C.A., Hadjipavlou-Litina D., Kefalas P. (2009). Natural and synthetic 2'-hydroxy-chalcones and aurones: Synthesis, characterization and evaluation of the antioxidant and soybean lipoxygenase inhibitory activity. Bioorgan. Med. Chem..

[30] Achenbach H., Waibel R., Addae-mansah I. (1983). Lignans and other constituents from *Carissa edulis*. Phytochemistry.

[31] Otsuka H., Takeuchi M., Inoshiri S., Sato T., Yamasaki K. (1989). Phenolic compounds from *Coix lachryma-jobi* var. Ma-Yuen. Phytochemistry.

[32] Freudenberg K., Schraube H. (1955). Sinapyl alcohol and the synthesis of syringaresinol. Chemische. Berichte..

[33] Li L.Y., Seeram N.P. (2010). Maple syrup phytochemicals include lignans, coumarins, a stilbene, and other previously unreported antioxidant phenolic compounds. J. Agric. Food Chem..

[34] Kobayashi M., Mahmud T., Yoshioka N., Hori K., Shibuya H., Kitagawa I. (1996). Indonesian medicinal plants. XVIII. Kompasinol A, a new stibeno-phenylpropanoid from the bark of *Koompassia malaccensis* (Fabaceae). Chem. Pharm. Bull..

[35] Lee D., Cuendet M., Vigo J.S., Graham J.G., Cabieses F., Fong H.H.S., Pezzuto J.M., Kinghorn A.D. (2001). A novel cyclooxygenase-inhibitory stilbenolignan from the seeds of *Aiphanes aculeata*. Org. Lett..

[36] Ha D.T., Chen Q.C., Hung T.M., Ui J.Y., Ngoc T.M., Thuong P.T., Kim H.J., Seong Y.H., Min B.S., Bae K.H. (2009). Stilbenes and oligostilbenes from leaf and stem of *Vitis amurensis* and their cytotoxic activity. Arch. Pharm. Res..

[37] Adam K.P. (1999). Phenolic constituents of the fern *Phegopteris connectilis*. Phytochemistry.

[38] Jin D.Z., Min Z.D., Chiou G.C.Y., Linuma M., Tanaka T. (1996). Two *p*-coumaroyl glycerides from *Juncus Effusus*. Phytochemistry.

[39] Luo J.G., Li L., Kong L.Y. (2012). Preparative separation of phenylpropenoid glycerides from the bulbs of *Lilium lancifolium* by high-speed counter-current chromatography and evaluation of their antioxidant activities. Food Chem..

[40] Fabrice V., Michel G. (1990). Enantiomeric purity of scytalone from different fungai sources. Tetrahedron.

[41] Limasset B., Doucen C., Dore J.C., Ojasoo T., Damon M., de Paulet A.C. (1993). Effects of flavonoids on the release of reactive oxygen species by stimulated human neutrophils. Multivariate analysis of structure-activity relationships (SAR). Biochem. Pharmacol..

[42] Satomi Y. (1993). Inhibitory effects of 3′-methyl-3-hydroxy-rchalcone on proliferation of human malignant tumor cells and on skin carcinogenesis. Int. J. Cancer.

[43] Lam S.H., Chen J.M., Kang C.J., Chen C.H., Lee S.S. (2008). α-Glucosidase inhibitors from the seeds of *Syagrus romanzoffiana*. Phytochemistry.

[44] Zhou J., Qu J., Shou G.X., Lü S.H. (2009). Determination of astilbin and engeletin in *Smilax glabra* Roxb. by RP-HPLC. Drug Stand. Chin..

[45] Yuan J.Z., Dou D.Q., Chen Y.J., Li W., Kazuo K., Tamotsu N., Yao X.S. (2004). Studies on dihydroflavonol glycosides from rhizome of *Smilax glabra*. Chin. J. Chin. Mater. Med..

[46] Yi Y.J., Cao Z.Z., Yang D.L., Cao Y., Wu Y.P., Zhao S.X. (1998). Studies on the chemical constituents of *Smilax glabra*. Acta Pharm. Sin..

[47] Chen G.Y., Shen L.S., Jiang P.F. (1996). Studies on flavanonol glucosides of *Smilax glabra* Roxb. Chin. J. Chin. Mater. Med..

[48] Wu B., Ma Y.P., Yuan J.Z., Sun Q.S. (2010). Isolation and identification of chemical constituents from rhizomes of *Smilax glabra* Roxb. J. Shenyang Pharm. Univ..

[49] Zhang M., Li H.T., Li Y. (1995). Studies on the chemical constituents of *Smilax glabra*. Chin. Med. Mat..

[50] Yim N.H., Ha D.T., Trung T.N., Kim J.P., Lee S.M., Na M.K., Jung H.J., Kim H.S., Kim Y.H., Bae K.H. (2010). The antimicrobial activity of compounds from the leaf and stem of *Vitis amurensis* against two oral pathogens. Bioorg. Med. Chem. Lett..

[51] National Committee for Clinical Laboratory Standard (2003). Methods for dilution antimicrobial susceptibility tests for bacteria that grow aerobically. Approved standard. NCCLS Document M7-A6.

[52] National Committee for Clinical Laboratory Standards (2002). Reference method for broth dilution antifungal susceptibility testing of yeasts. Approved Standard. NCCLS Document M27-A2.

