# Supplementary material for: Chemical Constituents from the Rhizomes of Smilax glabra and Their Antimicrobial Activity
Source: Molecules. 2013 May 8;18(5):5265–87. doi: 10.3390/molecules18055265 (PMC6270451; doi:10.3390/molecules18055265)
Supplement: Supplementary file 1 [file molecules-18-05265-s001.pdf]

Supplementary Materials

1. Spectroscopic Data of the Known Compounds

Taxifolin (**7**). Pale yellow needles; $[\alpha]_D^{20}$ +42.5 (*c* 1.30, acetone); 1H-NMR (400 MHz, CD_3OD) *δ*: 6.95 (1H, d, *J* = 1.2 Hz, H-2′), 6.83 (1H, dd, *J* = 8.0, 1.6 Hz, H-6′), 6.79 (1H, d, *J* = 8.0 Hz, H-5′), 5.87 (2H, s, H-6, 8), 4.90 (1H, d, *J* = 11.6 Hz, H-2), 4.49 (1H, d, *J* = 11.5 Hz, H-3); ^{13}C-NMR (100 MHz, CD_3OD)*δ*: 198.4 (C-4), 168.8 (C-7), 165.3 (C-5), 164.5 (C-9), 147.1 (C-4′), 146.3 (C-3′), 129.9 (C-1′), 120.9 (C-6′), 116.1 (C-2′), 115.9 (C-5′), 101.8 (C-10), 97.3 (C-6), 96.3 (C-8), 85.1 (C-2), 73.7 (C-3).

Naringenin (**8**). Pale yellow needles; ^{1}H-NMR (400 MHz, DMSO-*d*$_6$) *δ*: 7.30 (2H, d, *J* = 8.0 Hz, H-2′, 6′), 6.78 (2H, d, *J* = 8.0 Hz, H-3′, 5′), 5.87 (1H, s, H-6, 8), 5.43 (1H, dd, *J* = 12.4, 2.9 Hz, H-2), 3.28 (1H, dd, *J* = 13.2, 16.8 Hz, H-3b), 2.67 (1H, dd, *J* = 16.8, 2.9 Hz, H-3a); ^{13}C-NMR (100 MHz, DMSO-*d*$_6$) *δ*: 196.4 (C-4), 166.6 (C-7), 163.4 (C-5), 162.9 (C-9), 157.7 (C-4′), 128.8 (C-1′), 128.3 (C-2′, 6′), 115.1 (C-3′, 5′), 101.7 (C-10), 95.7 (C-6), 94.9 (C-8), 78.4 (C-2), 41.9 (C-3).

Dihydrokaempferol (**9**). White powder; $[\alpha]_D^{20}$ +22.8 (*c* 1.32, MeOH); ^{1}H-NMR (400 MHz, DMSO-*d*$_6$) *δ*: 11.89, 10.80, 9.53 (each 1H, s, OH), 7.30 (2H, d, *J* = 8.0 Hz, H-2′, 6′), 6.77 (2H, d, *J* = 7.6 Hz, H-3′, 5′), 5.90 (1H, d, *J* = 2.0 Hz, H-8), 5.85 (1H, d, *J* = 2.0 Hz, H-6), 5.74 (1H, d, *J* = 4.4 Hz, 3-OH), 5.04 (1H, d, *J* = 11.6 Hz, H-2), 4.57 (1H, dd, *J* = 10.4, 6.0 Hz, H-3); ^{13}C-NMR (100 MHz, DMSO-*d*$_6$) *δ*: 197.9 (C-4), 166.7 (C-7), 163.3 (C-5), 162.6 (C-9), 157.7 (C-4′), 129.4 (C-2′, 6′), 127.5 (C-1′), 114.9 (C-3′, 5′), 100.5 (C-10), 96.0 (C-6), 95.0 (C-8), 82.9 (C-2), 71.4 (C-3).

Sakuranetin (**10**). Pale yellow solid; $[\alpha]_D^{20}$ -7 (*c* 0.5, MeOH); ^{1}H-NMR (600 MHz, CD_3OD) *δ*: 7.31 (2H, d, *J* = 8.4 Hz, H-2′, 6′), 6.82 (2H, d, *J* = 8.4 Hz, H-3′, 5′), 6.06 (1H, d, *J* = 2.4 Hz, H-8), 5.89 (1H, s, H-6), 5.34 (1H, dd, *J* = 12.6, 3.0 Hz, H-2), 3.91 (3H, s, 7-OCH_3), 3.11(1H, dd, *J* = 17.4, 13.2 Hz, H-3b), 2.70 (1H, dd, *J* = 16.8, 3.0 Hz, H-3a); ^{13}C-NMR (150 MHz, CD_3OD) *δ*: 197.9 (C-4), 168.8 (C-7), 165.7 (C-5), 165.1 (C-9), 159.2(C-4′), 131.3 (C-1′), 129.2 (C-2′, 6′), 116.5 (C-3′, 5′), 103.5 (C-10), 96.4 (C-6), 93.9 (C-8), 80.7 (C-2), 57.1 (7-OCH_3), 44.2 (C-3).

Isoastilbin (**11**). White powder; $[\alpha]_D^{20}$ -144.6 (*c* 0.87, MeOH); ESIMS (*m/z*): 449 $[M-H]^-$, 473 $[M+Na]^+$; CD (6.35 × 10^{-5} MeOH): Δε (nm) +5.89 (342), -14.79 (300); ^{1}H-NMR (500 MHz, C_5D_5N) *δ*: 7.66 (1H, d, *J* = 1.5 Hz, H-2′), 7.30 (1H, dd, *J* = 8.0, 2.0 Hz, H-6′), 7.25 (1H, d, *J* = 8.5 Hz, H-5′), 6.40 (1H, d, *J* = 2.0 Hz, H-6), 6.39 (1H, d, *J* = 2.0 Hz, H-8), 5.88 (1H, d, *J* = 1.5 Hz, H-2), 5.81 (1H, d, *J* = 2.5Hz, H-3), 4.91 (1H, d, *J* = 2.5 Hz, H-1″), 4.51 (1H, br. s, H-2″), 4.37 (1H, dd, *J* = 9.5, 3.5 Hz, H-5″), 4.14 (1H, m, H-3″), 3.41 (1H, m, H-4″), 1.45 (3H, d, *J* = 6.0 Hz, H-6″); ^{13}C-NMR (125 MHz, C_5D_5N) *δ*: 194.2 (C-4), 168.9 (C-7), 165.6 (C-5), 163.7 (C-9), 147.6 (C-4′), 147.4 (C-3′), 127.6 (C-1′), 118.9 (C-6′), 116.4 (C-5′), 115.5 (C-2′), 101.5(C-1″), 100.6 (C-10), 97.5 (C-6), 96.4 (C-8), 81.7 (C-2), 74.4 (C-3), 73.2 (C-4″), 72.4 (C-3″), 71.9 (C-2″), 70.4 (C-5″), 18.4 (C-6″).

Astilbin (**12**). White powder; $[\alpha]_D^{20}$ -13.5 (*c* 0.76, MeOH); ESIMS (*m/z*): 449 $[M-H]^-$, 473 $[M+Na]^+$; CD (3.98 × 10^{-5} MeOH): Δε (nm) +5.89-15.46 (294), +5.66 (330); ^{1}H-NMR (500 MHz, CD_3OD) *δ*: 6.95 (1H, d, *J* = 2.0 Hz, H-2′), 6.82 (1H, dd, *J* = 8.5, 2.0 Hz, H-6′), 6.80 (1H, d, *J* = 8.0 Hz, H-5′), 5.91 (1H, d, *J* = 2.5 Hz, H-6), 5.89 (1H, d, *J* = 2.5 Hz, H-8), 5.07 (1H, d, *J* = 10.5 Hz, H-2), 4.55 (1H, d,

J = 10.5 Hz, H-3), 4.24 (1H, dq, J = 10.0, 6.5 Hz, H-5″), 4.04 (1H, s, H-1″), 3.65 (1H, dd, J = 9.5, 3.5 Hz, H-3″), 3.53 (1H, br. s, H-2″), 3.31 (1H, dd, J = 9.6, 9.6 Hz, H-4″), 1.17 (1H, d, J = 6.0 Hz, H-6″); ^{13}C-NMR (125 MHz, CD_3OD) δ: 196.0 (C-4), 168.6 (C-7), 165.5 (C-5), 164.1 (C-9), 147.4 (C-4′), 146.6 (C-3′), 129.2 (C-1′), 120.5 (C-6′), 116.3(C-5′), 115.5(C-2′), 102.5 (C-1″), 102.2 (C-10), 97.4 (C-6), 96.3 (C-8), 83.9 (C-2), 78.6 (C-3), 73.8 (C-4″), 72.2 (C-3″), 71.8 (C-2″), 70.5 (C-5″), 17.9(C-6″).

Neoastilbin (**13**). White solid; $[\alpha]_D^{20}$ -85.6 (*c* 0.85, MeOH); CD (2.05 × 10^{-5} MeOH): Δε (nm) +0.76 (297), -1.43 (330); ^{1}H-NMR (600 MHz, CD_3OD) δ: 6.97 (1H, s, H-2′), 6.80 (2H, s, H-5′, 6′), 5.92 (1H, d, J = 2.4 Hz, H-6), 5.89 (1H, d, J = 2.4 Hz, H-8), 5.16 (1H, s, H-2), 4.99(1H, s, H-1″), 4.64 (1H, d, J = 11.4 Hz, H-3), 4.00 (1H, s, H-2″), 3.38 (1H, dd, J = 9.6, 3.0 Hz, H-3″), 3.20 (1H, t, H-4″), 2.31 (1H, m, H-5″), 0.90 (3H, d, J = 6.0 Hz, H-6″); ^{13}C-NMR (150 MHz, CD_3OD) δ: 197.9 (C-4), 168.9 (C-7), 165.7 (C-5), 164.5 (C-9), 147.6 (C-4′), 146.8 (C-3′), 130.2 (C-1′), 121.2 (C-6′), 116.5 (C-5′), 115.7 (C-2′), 103.0 (C-1″), 102.3 (C-10), 97.6 (C-6), 96.5 (C-8), 83.9 (C-2), 77.1 (C-3), 73.6 (C-4″), 72.1 (C-3″), 72.1 (C-2″), 70.5 (C-5″), 18.1 (C-6″).

Neoisoastilbin (**14**). White solid; ESIMS (*m/z*): 449 $[M-H]^-$, 473 $[M+Na]^+$; CD (5.05 × 10^{-5} MeOH): Δε (nm) +2.47 (297), -1.49 (340); ^{1}H-NMR (500 MHz，CD_3OD) δ: 6.99 (1H, d, J = 2.0 Hz, H-2′), 6.84 (1H, dd, J = 6.0, 2.0 Hz, H-6′), 6.79 (1H, d, J = 8.5 Hz, H-5′), 5.95 (1H, d, J = 2.5 Hz, H-6), 5.92 (1H, d, J = 2.5 Hz, H-8), 5.32 (1H, d, J = 1.5 Hz, H-2), 4.23 (1H, d, J = 1.5 Hz, H-1″), 4.06 (1H, d, J = 2.0 Hz, H-3), 3.59 (1H, s, H-2″), 3.49 (1H, dq, J = 9.0, 6.5 Hz, H-5″), 3.34 (1H, s, H-3″), 3.26 (1H, s, H-4″), 1.13 (3H, d, J = 6.5 Hz, H-6″); ^{13}C-NMR (125 MHz, CD_3OD)δ: 193.9 (C-4), 168.7 (C-7), 166.0 (C-5), 164.5 (C-9), 146.7 (C-4′), 146.3 (C-3′), 128.5 (C-1′), 119.4 (C-6′), 116.1 (C-5′), 115.3 (C-2′), 102.6 (C-1″), 102.1 (C-10), 97.2 (C-6), 96.0 (C-8), 82.3 (C-2), 78.4 (C-3), 73.7 (C-4″), 72.0 (C-3″), 71.6 (C-2″), 70.6 (C-5″), 17.5 (C-6″).

Engeletin (**15**). Yellow powder; $[\alpha]_D^{20}$ -14.2 (*c* 0.32, MeOH); ^{1}H-NMR (500 MHz, CD_3OD) δ: 7.35 (2H, br. d, J = 8.5 Hz, H-2′, 6′), 6.83 (2H, br .d, J = 8.5 Hz, H-3′, 5′), 5.91 (1H, d, J = 2.5 Hz, H-8), 5.89 (1H, d, J = 2.5 Hz, H-6), 5.15 (1H, d, J = 9.5 Hz, H-2), 4.60 (1H, d, J = 11.0 Hz, H-3), 4.00 (1H, d, J = 1.5 Hz, H-1″), 3.64 (1H, dd, J = 3.0, 1.5 Hz, H-2″), 3.49 (1H, dd, J = 9.5, 3.0 Hz, H-3″), 3.29 (1H, m, H-4″), 2.25 (1H, m, H-5″), 1.17 (3H, d, J = 6.5 Hz, H-6″); ^{13}C-NMR (125 MHz, CD_3OD) δ: 196.0 (C-4), 168.7 (C-7), 165.5 (C-5), 164.1 (C-9), 159.5 (C-4′), 130.2 (C-2′, 6′), 128.6(C-1′), 116.4 (C-3′, 5′), 102.5 (C-1″), 102.2 (C-10), 97.4 (C-8), 96.3 (C-6), 83.9 (C-2), 78.7 (C-3), 73.8 (C-4″), 72.2 (C-3″), 70.5 (C-5″), 18.0 (C-6″).

Arthromerin B (**16**). Pale yellow powder; $[\alpha]_D^{20}$ +28.3 (*c* 0.90, MeOH); CD (2.16 × 10^{-5} MeOH): Δε (nm) -19.41 (291), +3.99 (330); ^{1}H-NMR (500 MHz, CD_3OD) δ: 7.35 (2H, d, J = 8.5 Hz, H-2′, 6′), 6.78 (2H, d, J = 8.5 Hz, H-3′, 5′), 5.92 (1H, d, J = 2.0 Hz, H-8), 5.89 (1H, d, J = 1.5 Hz, H-6), 5.18 (1H, d, J = 11.0 Hz, H-2), 4.84 (1H, d, J = 8.5 Hz, H-3), 4.70 (1H, d, J = 7.4 Hz, H-1″), 3.89 (1H, dd, J = 12.0, 2.4 Hz, H-6″b), 3.76 (1H, dd, J = 12.0, 6.0 Hz, H-6″a), 3.55 (1H, m, H-2″), 3.52 (1H, m, H-5″), 3.35 (1H, m, H-3″), 3.29 (1H, m, H-4″); ^{13}C-NMR (125 MHz, CD_3OD) δ: 196.3 (C-4), 169.2 (C-7), 165.5 (C-5), 164.4(C-9), 159.5 (C-4′), 130.3 (C-2′, 6′), 128.4 (C-1′), 116.4 (C-3′, 5′), 102.4

(C-1″), 101.5 (C-10), 97.4 (C-8), 96.4 (C-6), 83.7 (C-2), 77.6 (C-5″), 76.4 (C-3), 73.1 (C-3″), 71.1 (C-2″), 66.9 (C-4″), 63.5 (C-6″).

Sinensin (**17**). Yellow solid; CD (2.10×10^{-5} MeOH): Δε (nm) -4.58 (298), +1.17 (336); ESIMS (*m/z*): 449 $[M-H]^-$, 473 $[M+Na]^+$; ^{1}H-NMR (600 MHz, CD_3OD) *δ*: 7.35 (2H, d, *J* = 8.4 Hz, H-2′, 6′), 6.83 (2H, d, *J* = 8.5 Hz, H-3′, 5′), 6.47 (1H, d, *J* = 2.4 Hz, H-8), 6.09 (1H, d, *J* = 1.8 Hz, H-6), 4.87 (1H, d, *J* = 11.4 Hz, H-2), 4.39 (1H, d, *J* = 11.4 Hz, H-3), 4.80 (1H, d, *J* = 7.4Hz, H-1″), 3.94 (1H, dd, *J* = 12.0, 1.8 Hz, H-6″b), 3.76 (1H, dd, *J* = 12.0, 5.4 Hz, H-6″a), 3.45-3.58 (4H, m, H-2″-5″); ^{13}C-NMR (150 MHz, CD_3OD) *δ*: 193.9 (C-4), 167.5 (C-5), 166.1 (C-9), 162.2 (C-7), 159.4 (C-4′), 130.5 (C-2′, 6′), 129.5 (C-1′), 116.3 (C-3′, 5′), 105.1(C-1″), 104.4 (C-10), 100.2 (C-8), 99.3 (C-6), 84.5 (C-2), 78.8 (C-5″), 77.6(C-3), 74.8 (C-3″), 71.4 (C-2″), 67.0 (C-4″), 62.7 (C-6″).

(2R, 3R)-Taxifolin 3′-O-β-D-glucopyranoside (**18**). Pale yellow solid; CD (2.20×10^{-5} MeOH): Δε (nm) -4.89 (292), +0.45 (326); ^{1}H-NMR (600 MHz, CD_3OD) *δ*: 7.38 (1H, d, *J* = 1.8 Hz, H-2′), 7.10 (1H, dd, *J* = 8.4, 2.4 Hz, H-6′), 6.90 (1H, d, *J* = 7.8 Hz, H-5′), 5.93 (1H, d, *J* = 2.4 Hz, H-6), 5.89 (1H, d, *J* = 2.4 Hz, H-8), 4.98 (1H, d, *J* = 11.4 Hz, H-2), 4.83 (1H, d, *J* = 7.4 Hz, H-1″), 4.57 (1H, d, *J* = 11.4 Hz, H-3), 3.89 (1H, dd, *J* = 12.0, 2.4 Hz, H-6″), 3.68 (1H, dd, *J* = 12.0, 6.0 Hz, H-2″), 3.64 (1H, s, H-5″), 3.51 (1H, dd, *J* = 9.6, 10.2 Hz, H-3″), 3.43 (1H, m, H-4″); ^{13}C-NMR (150 MHz, CD_3OD) *δ*: 198.8 (C-4), 168.7 (C-7), 164.8 (C-5), 164.0 (C-9), 149.3 (C-4′), 146.9 (C-3′), 129.7 (C-1′), 124.9 (C-6′), 118.5 (C-2′), 117.1 (C-5′), 104.3 (C-1″), 101.2 (C-10), 97.6 (C-6), 96.5 (C-8), 85.1 (C-2), 78.6 (C-5″), 77.9 (C-3″), 75.1 (C-2″), 73.7 (C-3), 71.7 (C-4″), 62.8 (C-6″).

(2S,3S)-Glucodistylin (**19**). Pale yellow solid; CD (3.62×10^{-5} MeOH): Δε (nm) +1.51 (295), -3.24 (327); ^{1}H-NMR (600 MHz, CD_3OD) *δ*: 6.95 (1H, d, *J* = 1.8 Hz, H-2′), 6.79 (1H, dd, *J* = 8.0, 1.8Hz, H-6′), 6.75 (1H, d, *J* = 8.4Hz, H-5′), 5.90 (1H, d, *J* = 1.2 Hz, H-6), 5.89 (1H, d, *J* = 1.8 Hz, H-8), 5.26 (1H, d, *J* = 9.0 Hz, H-2), 4.90 (1H, d, *J* = 7.4 Hz, H-1″), 4.68 (1H, d, *J* = 7.8Hz, H-3), 3.80 (1H, dd, *J* = 12.0, 2.4 Hz, H-6″b), 3.70 (1H, dd, *J* = 12.0, 6.0 Hz, H-6″a), 3.43 (1H, m, H-2″), 3.35 (1H, s, H-5″), 3.19 (1H, m, H-3″), 3.13 (1H, m, H-4″); ^{13}C-NMR (150 MHz, CD_3OD) *δ*: 195.9 (C-4), 170.1 (C-7), 166.9 (C-5), 165.9 (C-9), 147.5 (C-3′), 146.3 (C-3′), 129.3 (C-1′), 121.3 (C-6′), 116.4 (C-2′), 116.2 (C-5′), 104.8 (C-1″), 102.7 (C-10), 97.7 (C-6), 96.5 (C-8), 83.7 (C-2), 78.1 (C-5″), 77.9 (C-3″), 75.6 (C-2″), 72.5 (C-3), 71.7 (C-4″), 63.1 (C-6″).

(-)-Epicatechin (**20**). White powder; $[\alpha]_D^{20}$ -70.50 (*c* 0.20, MeOH); ^{1}H-NMR (400 MHz, DMSO-d_6) *δ*: 6.88 (1H, s, H-2′), 6.65 (1H, d, *J* = 8.0 Hz, H-6′), 6.64 (1H, d, *J* = 8.0 Hz, H-5′), 5.88 (1H, d, *J* = 2.4 Hz, H-8), 5.70 (1H, d, *J* = 2.0 Hz, H-6), 4.64 (1H, s, H-2), 3.98 (1H, m, H-3), 2.67 (1H, dd, *J* = 16.4, 4.4 Hz, H-4a), 2.44 (1H, dd, *J* = 16.4, 3.6 Hz, H-4b); ^{13}C-NMR (100 MHz, DMSO-d_6) *δ*: 156.9 (C-9), 156.7 (C-7), 156.2 (C-5), 144.9 (C-3′), 144.8 (C-4′), 131.0 (C-1′), 118.4 (C-6′), 115.3 (C-5′), 115.2 (C-2′), 98.9 (C-10), 95.5 (C-6), 94.5 (C-8), 78.5 (C-2), 65.3 (C-3), 28.6 (C-4).

(+)-Catechin (**21**). White powder; $[\alpha]_D^{20}$ +25.5 (*c* 0.08, MeOH); ^{1}H-NMR (400 MHz, DMSO-d_6) *δ*: 6.70 (1H, d, *J* = 2.0 Hz, H-2′), 6.67 (1H, dd, *J* = 8.0, 1.6 Hz, H-6′), 6.58 (1H, d, *J* = 6.4 Hz, H-5′), 5.87 (1H, d, *J* = 2.0 Hz, H-8), 5.67 (1H, d, *J* = 2.0 Hz, H-6), 4.46 (1H, d, *J* = 7.6 Hz, H-2), 3.79 (1H, m, H-3), 2.64 (1H, dd, *J* = 16.0, 5.2 Hz, H-4a), 2.33 (1H, dd, *J* = 16.4, 7.6 Hz, H-4b); ^{13}C-NMR (100

MHz, DMSO-d_6) *δ*: 156.4 (C-7), 156.1 (C-5), 155.3 (C-9), 144.8 (C-3′, 4′), 130.6 (C-1′), 118.4 (C-6′), 115.0 (C-5′), 114.5 (C-2′), 99.0 (C-10), 95.1 (C-6), 93.8 (C-8), 81.0 (C-2), 66.3 (C-3), 27.8 (C-4).

Cinchonain Ib (**22**). Pale yellow solid; CD (6.20×10^{-4} MeOH): Δε (nm) +7.12 (232); ESIMS (*m/z*): 451 $[M-H]^-$, 488 $[M+Na]^+$; ^{1}H-NMR (600 MHz, CD_3OD) *δ*: 6.83 (1H, d, *J* = 1.8 Hz, H-2′), 6.69 (1H, d, *J* = 7.8 Hz, H-5″), 6.67 (1H, dd, *J* = 8.0, 2.0 Hz, H-6′), 6.63 (1H, d, *J* = 8.0 Hz, H-5′), 6.61 (1H, d, *J* = 1.8 Hz, H-2″), 6.54 (1H, dd, *J* = 8.2, 2.0 Hz, H-6″), 6.21 (1H, s, H-6), 4.98 (1H, s, H-2), 4.46 (1H, br. d, *J* = 5.4 Hz, H-7″), 4.21 (1H, m, H-3), 3.01 (1H, dd, *J* = 15.6, 7.2 Hz, H-8″a), 2.94 (1H, dd, *J* = 16.8, 4.2 Hz, H-4a), 2.87 (1H, dd, *J* = 16.2, 1.8 Hz, H-8″b), 2.83 (1H, dd, *J* = 17.4, 2.4Hz, H-4b); ^{13}C-NMR (150 MHz, CD_3OD) *δ*: 170.7 (C-9″), 157.3 (C-5), 153.5 (C-9), 152.0 (C-7), 146.4 (C-3″), 145.9 (C-4′), 145.8 (C-4″), 145.3 (C-3′), 135.3 (C-1″), 131.7 (C-1′), 119.4 (C-6′), 119.3 (C-6″), 116.5 (C-5″), 115.9 (C-5′), 115.3 (C-2″), 115.0 (C-2′), 106.2 (C-8), 105.3 (C-10), 96.4 (C-6), 79.9 (C-2), 67.0 (C-3), 38.3 (C-8″), 35.2 (C-7″), 29.3 (C-4).

Cinchonain Ia (**23**). Pale yellow solid; CD (3.5×10^{-4} MeOH): Δε(nm) -12.48 (235); ESIMS (*m/z*): 451 $[M-H]^-$, ^{1}H-NMR (600 MHz, CD_3OD) *δ*: 6.97 (1H, d, *J* = 1.8 Hz, H-2′), 6.78 (1H, dd, *J* = 8.0, 1.8 Hz, H-6′), 6.77 (1H, d, *J* = 8.0 Hz, H-5′), 6.61 (1H, d, *J* = 8.2 Hz, H-6″), 6.52 (1H, d, *J* = 1.8 Hz, H-2″), 6.45 (1H, dd, *J* = 7.8, 1.8 Hz, H-5″), 6.21 (1H, s, H-6), 4.87 (1H, s, H-2), 4.55 (1H, br. d, *J* = 6.0 Hz, H-7″), 4.25 (1H, m, H-3), 3.07 (1H, dd, *J* = 15.6, 7.2 Hz, H-8″a), 2.91 (1H, dd, *J* = 16.8, 4.2 Hz, H-4a), 2.87 (1H, dd, *J* = 15.6, 2.0 Hz, H-8″b), 2.84 (1H, dd, *J* = 16.8, 2.0 Hz, H-4b); ^{13}C-NMR (150 MHz, CD_3OD) *δ*: 170.7 (C-9″), 157.3 (C-5), 153.4 (C-9), 152.1 (C-7), 146.3 (C-3″), 146.0 (C-4′), 145.8 (C-4″), 145.1 (C-3′), 135.4 (C-1″), 131.9 (C-1′), 119.2 (C-6′), 119.2 (C-6″), 116.5 (C-5″), 116.0 (C-5′), 115.1 (C-2″), 115.1 (C-2′), 106.0 (C-8), 105.2 (C-10), 96.2 (C-6), 79.7 (C-2), 66.6 (C-3), 38.6 (C-8″), 35.4 (C-7″), 29.5 (C-4).

Apigenin (**24**). Yellow powder; ESIMS (*m/z*): 269 $[M-H]^-$, 271 $[M+H]^+$; ^{1}H-NMR (500 MHz, DMSO-d_6) *δ*: 10.03 (1H, s, 4′-OH), 7.73 (2H, d, *J* = 8.5 Hz, H-2′, 6′), 6.84 (2H, d, *J* = 8.5 Hz, H-3′, 5′), 6.77 (1H, s, H-3), 6.51 (1H, d, *J* = 2.0 Hz, H-8), 6.16 (1H, d, *J* = 2.0 Hz, H-6); ^{13}C-NMR (125 MHz, DMSO-d_6) *δ*: 179.0 (C-4), 167.5 (C-2), 167.1 (C-7), 163.7 (C-5), 163.0 (C-4′), 158.7(C-9), 132.7 (C-2′, 6′), 123.3 (C-1′), 115.9 (C-3′, 5′), 105.1 (C-10), 104.3 (C-3), 97.7 (C-6), 93.9 (C-8).

Quercetin (**25**). Yellow powder; ^{1}H-NMR (600 MHz, DMSO-d_6) *δ*: 12.48 (s, 1H, C5-OH), 10.78 (s, 1H, C7-OH), 9.58 (s, 1H, C4′-OH), 9.35 (s, 1H, 3-OH), 9.29 (s, 1H, C3′-OH), 7.66 (1H, d, *J* = 1.2 Hz, H-2′), 7.53 (1H, dd, *J* = 8.4, 1.8 Hz, H-6′), 6.87 (1H, d, *J* = 8.4 Hz, H-5′), 6.39(1H, d, *J* = 1.8 Hz, H-8), 6.17(1H, d, *J* = 1.8 Hz, H-6); ^{13}C-NMR (150 MHz, DMSO-d_6) *δ*: 175.8 (C-4), 163.9 (C-7), 160.7 (C-5), 156.1 (C-9), 147.7 (C-4′), 146.8 (C-2), 145.0 (C-3′), 135.7 (C-3), 121.9 (C-1′), 119.9 (C-6′), 115.6 (C-5′), 115.0 (C-2′), 102.9 (C-10), 98.2 (C-6), 93.3(C-8).

Luteolin (**26**). Yellow powder; ESIMS (*m/z*): 285 $[M-H]^-$, 287 $[M+H]^+$; ^{1}H-NMR (600 MHz, CD_3OD) *δ*: 7.48 (1H, d, *J* = 1.8 Hz, H-2′), 7.19 (1H, dd, *J* = 8.4, 1.8 Hz, H-6′), 6.83 (1H, d, *J* = 8.4 Hz, H-5′), 6.73 (1H, s, H-3), 6.57 (1H, br. s, H-8), 6.21 (1H, br. s, H-6); ^{13}C-NMR (150 MHz, CD_3OD) *δ*: 182.9 (C-4), 169.6 (C-7), 169.5 (C-2), 166.5 (C-5), 159.9 (C-9), 148.9 (C-4′), 147.9 (C-3′), 125.9 (C-1′), 125.7 (C-6′), 118.6 (C-5′), 116.6 (C-2′), 104.7 (C-10), 103.6 (C-3), 98.5 (C-6), 91.6 (C-8).

Myricetin (**27**). Yellow powder; ^{1}H-NMR (600 MHz, CD_3OD) δ: 6.97 (2H, s, H-2′, 6′), 6.49 (1H, s, H-8), 6.20 (1H, d, J = 1.8Hz, H-6); ^{13}C-NMR (150 MHz, CD_3OD) δ: 182.8 (C-4), 169.7 (C-7), 169.5 (C-5), 159.9 (C-9), 148.0 (C-2), 147.0 (C-3′, 5′), 137.2 (C-3), 136.5 (C-4′), 124.6 (C-1′), 111.8 (C-2′, 6′), 104.7 (C-10), 99.3 (C-6), 91.6 (C-8).

Kukulkanin B (**28**). Yellow solid; ESIMS (*m/z*): 285 $[M-H]^-$, 287 $[M+H]^+$; ^{1}H-NMR (500 MHz, CD_3OD) δ: 7.98 (1H, d, J = 9.5 Hz, H-6'), 7.96 (2H, d, J = 8.5 Hz, H-2, 6), 7.64 (1H, d, J = 16.0 Hz, H-β), 7.22 (1H, d, J = 9.0 Hz, H-α), 6.88 (2H, d, J = 8.5 Hz, H-3, 5), 6.64 (1H, d, J = 9.0 Hz, H-5'), 3.84 (3H, s, 3′-OCH_3); ^{13}C-NMR (125 MHz, CD_3OD) δ: 191.3 (C=O), 163.7 (C-4′), 150.8 (C-4), 149.9 (C-3′), 141.1 (C-β), 139.7 (C-2′), 132.2 (C-2, 6), 131.3 (C-6′), 121.5 (C-1), 120.5 (C-α), 120.3 (C-3, 5), 116.4 (C-1′), 112.7 (C-5′), 61.8 (3′-OCH_3).

4,4′,6-Trihydroxyaurone (**29**). Yellow powder; ESIMS (*m/z*): 269 $[M-H]^-$, 271 $[M+H]^+$; ^{1}H-NMR (500 MHz, DMSO-d_6) δ: 10.83 (1H, s, OH), 10.79 (1H, s, OH), 10.00 (1H, s, OH), 7.74 (2H, d, J = 8.5 Hz, H-2′, 6′), 6.85 (2H, d, J = 9.0 Hz, H-3′, 5′), 6.53 (1H, s, -CH=), 6.19 (1H, d, J = 2.0 Hz, H-7), 6.05 (1H, d, J = 1.5 Hz, H-5); ^{13}C-NMR (125 MHz, DMSO-d_6) δ: 179.0 (C=O), 167.5 (C-4, 6), 158.7 (C-8), 158.1 (C-4′), 150.0 (C-2), 132.7 (C-2′, 6′), 123.3 (C-1′), 115.9 (C-3′, 5′), 109.0 (C-10), 102.8 (C-9), 97.6 (C-5), 90.4 (C-7).

Aureusidin (**30**). Yellow powder; ESIMS (*m/z*): 285 $[M-H]^-$, 287 $[M+H]^+$; ^{1}H-NMR (400 MHz, CD_3OD) δ: 7.49 (1H, d, J = 1.2 Hz, H-2′), 7.21 (1H, dd, J = 8.0, 1.2 Hz, H-6′), 6.85 (1H, d, J = 8.0 Hz, H-5′), 6.59 (1H, s, -CH=), 6.22 (1H, br. s, H-5), 6.02 (1H, br. s, H-7); ^{13}C-NMR (100 MHz, CD_3OD) δ: 182.9 (C=O), 169.6 (C-6), 169.4 (C-4), 160.0 (C-8), 148.9 (C-4′), 147.9 (C-2), 146.6 (C-3′), 125.9 (C-1′), 125.8 (C-6′), 118.7 (C-2′), 116.6 (C-5′), 112.8 (C-10), 104.8 (C-9), 98.5 (C-7), 91.6 (C-5).

(-)-Secoisolariciresinol (**31**). White powder; $[\alpha]_D^{20}$ -35.1 (*c* 0.20, MeOH); ESIMS (*m/z*): 385 $[M+Na]^+$; ^{1}H-NMR (400MHz, CD_3OD) δ: 6.65 (2H, d, J = 8.0 Hz, H-5, 5′), 6.58 (2H, d, J = 1.6 Hz, H-2, 2′), 6.53 (2H, dd, J = 8.0, 2.0 Hz, H-6, 6′), 3.73 (6H, s, 3, 3′-OCH_3), 3.58 (4H, m, H-9, 9′), 2.54 (2H, dd, J = 13.6, 7.6 Hz, H-7, 7′), 2.66 (2H, dd, J = 13.6, 7.6 Hz, H-7, 7′), 1.90 (2H, m, H-8, 8′); ^{13}C-NMR (100 MHz, DMSO-d_6) δ: 147.2 (C-3, 3′), 144.3 (C-4, 4′), 132.2 (C-1, 1′), 121.1 (C-6, 6′), 115.0 (C-5, 5′), 112.9 (C-2, 2′), 60.2 (C-9, 9′), 55.4 (3, 3′-OCH_3), 42.4 (C-8, 8′), 33.9 (C-7, 7′).

4-Ketopinoresinol (**32**). Colorless solid; ESIMS (*m/z*): 371 $[M-H]^-$, 395 $[M+Na]^+$; ^{1}H-NMR (600 MHz, CD_3OD) δ: 6.78–6.97 (6H, m, ArH), 5.39 (1H, d, J = 4.2 Hz, H-6), 5.23 (1H, d, J = 4.2 Hz, H-2), 4.29 (1H, dd, J = 9.6, 7.2 Hz, H-8′), 4.03 (1H, dd, J = 9.6, 4.8 Hz, H-8), 3.87 (6H, s, 2 × OCH_3), 3.66 (2H, m, H-1, 5); ^{13}C-NMR (150 MHz, CD_3OD) δ: 179.9 (C-4), 149.6 (C-4″), 149.4 (C-4′), 148.4 (C-3″), 147.7 (C-3′), 133.4 (C-1″), 132.6 (C-1′), 119.9 (C-6″), 119.7 (C-6′), 116.6 (C-5″), 116.4 (C-5′), 110.8 (C-2″), 109.9 (C-2′), 85.3 (C-6), 82.6 (C-2), 74.0 (C-8), 56.7 (OCH_3), 56.6 (OCH_3), 51.2 (C-1), 46.8 (C-5).

1,4-Bis(4-hydroxy-3,5-dimethoxyphenyl)-2,3-bis(hydroxymethyl)-1,4-butanediol (Smiglabranol) (**33**). Pale yellow solid; EIMS *m/z*: 418 $[M-2H_2O]^+$, 319 (100), 210 (50), 226(41), 320(20), 436 (15); ^{1}H-NMR (500 MHz, CD_3OD) δ: 6.74 (4H, s, H-2, 2′, 6, 6′), 4.95 (2H, d, J = 8.5 Hz, H-7, 7′), 3.86

(12H, s, 4 × -OCH_3), 3.71 (2H, dd, *J* = 8.5, 2.5 Hz, H-9b, 9′b), 3.62 (2H, dd, *J* = 11.5, 5.0 Hz, H-9a, 9′a), 2.31 (2H, m, H-8, 8′); ^{13}C-NMR (125 MHz, CD_3OD) *δ*: 149.3 (C-3, 3′, 5, 5′), 136.2 (C-1, 1′), 134.2 (C-4, 4′), 104.8 (C-2, 2′, 6, 6′), 84.6 (C-7, 7′), 61.7 (C-9, 9′), 56.8 (C-8, 8′), 55.2 (3, 3′-OCH_3).

(+)-Lyoniresinol (**34**). Colorless solid; ESIMS (*m/z*): 419 $[M-H]^-$, 443 $[M+Na]^+$; ^{1}H-NMR (600 MHz, CD_3OD) *δ*: 6.59 (1H, s, H-6), 6.38 (2H, s, H-2′, 6′), 4.31 (1H, d, *J* = 5.4 Hz, H-7′), 3.87 (3H, s, 5-OCH_3), 3.74 (6H, s, 3′, 5′-OCH_3), 3.60 (2H, m, H-9b, 9b′), 3.50 (2H, d, *J* = 5.4 Hz, H-9a, 9a′), 3.38 (3H, s, 3-OCH_3), 2.70 (1H, dd, *J* = 4.8, 15.0 Hz, H-7b), 2.57 (1H, dd, *J* = 11.4, 14.4 Hz, H-7a), 1.97 (1H, m, H-8′), 1.63 (1H, m, H-8); ^{13}C-NMR (150 MHz, CD_3OD) *δ*: 149.2 (C-3′, 5′), 148.9 (C-5), 147.9 (C-3), 139.5 (C-4, 1′), 134.8 (C-4′), 130.4 (C-1), 126.5 (C-2), 108.0 (C-6), 107.1 (C-2′, 6′), 67.0 (C-9), 64.4 (C-9′), 60.4 (3-OCH_3), 57.0 (3′, 5′-OCH_3), 56.8 (5-OCH_3), 49.8(C-8′)， 42.5 (C-7′), 41.1 (C-8), 33.8 (C-7).

Kompasinol A (**35**). Pale yellow solid; ESIMS (*m/z*): 451 $[M-H]^-$, 453 $[M+H]^+$; ^{1}H-NMR (600 MHz, CD_3OD) *δ*: 6.85 (1H, d, *J* = 1.8 Hz, H-10), 6.78 (1H, d, *J* = 7.8 Hz, H-13), 6.75(1H, dd, *J* = 7.8, 1.8 Hz, H-14), 6.33(1H, s, H-2′), 6.33 (1H, s, H-6′), 6.24 (1H, d, *J* = 1.7 Hz, H-6), 6.19 (1H, d, *J* = 1.8 Hz, H-4), 4.65 (1H, d, *J* = 4.2 Hz, H-8), 4.47 (1H, dd, *J* = 8.4, 7.8 Hz, H-9′a), 4.13 (1H, br. s, H-7′), 3.77 (1H, m, H-7), 3.73 (6H, s, 3′, 5′-OCH_3), 3.54 (1H, t, *J* = 8.6 Hz, H-9′b), 3.04 (1H, ddd, *J* = 8.4, 8.4, 1.2 Hz, H-8′); ^{13}C-NMR (150 MHz, CD_3OD) *δ*: 160.5 (C-5), 156.3 (C-3), 149.1 (C-3′, 5′), 148.5 (C-1), 146.5 (C-12), 146.0 (C-11), 137.9 (C-1′), 135.2 (C-9), 134.6 (C-4′), 123.0 (C-2), 119.1 (C-14), 116.3 (C-13), 114.5 (C-10), 105.4 (C-2′, 6′), 103.2 (C-6), 102.8 (C-4), 89.4(C-8), 75.0 (C-9′), 59.8 (C-7), 56.6 (3′, 5′-OCH_3), 56.5 (C-8′), 52.1 (C-7′).

Aiphanol (**36**). Pale yellow powder; ESIMS (*m/z*): 451 $[M-H]^-$; ^{1}H-NMR (600 MHz, CD_3OD) *δ*: 7.09 (1H, s, H-8), 7.04 (1H, d, *J* = 8.4 Hz, H-6), 6.93 (1H, d, *J* = 15.0 Hz, H-10), 6.83 (1H, d, *J* = 16.2 Hz, H-9), 6.78 (1H, d, *J* = 8.0 Hz, H-5), 6.75 (2H, s, H-2′), 6.45 (2H, s, H-12), 6.17 (1H, s, H-14), 4.87 (1H, d, *J* = 8.0 Hz, H-2), 4.08 (1H, m, H-3), 3.87 (6H, s, 3′, 5′-OCH_3), 3.71 (1H, d, *J* = 12.0 Hz, CH_2OH), 3.49 (1H, dd, *J* = 12.0, 4.2 Hz, CH_2OH); ^{13}C-NMR (150 MHz, CD_3OD) *δ*: 159.7 (C-13), 149.4 (C-3′), 145.4 (C-8a), 144.7 (C-4a), 141.0 (C-11), 137.2 (C-4′), 132.5 (C-7), 129.0 (C-10), 128.6 (C-1′), 128.4 (C-9), 121.1 (C-6), 118.1 (C-5), 115.6 (C-8), 105.9 (C-2′), 105.9 (C-12), 102.9 (C-14), 80.1 (C-3), 78.0 (C-2), 62.1 (CH_2OH), 56.8 (3′, 5′-OCH_3).

Trans-resveratrol (**37**). White powder; ^{1}H-NMR (600 MHz, CD_3OD) *δ*: 7.35 (2H, d, *J* = 8.4 Hz, H-2′, 6′), 6.95 (1H, d, *J* = 16.2 Hz, H-8), 6.80 (1H, d, *J* = 16.8 Hz, H-7), 6.76 (2H, d, *J* = 8.4 Hz, H-3′, 5′), 6.45 (2H, d, *J* = 1.8 Hz, H-2, 6), 6.16 (1H, t, H-4); ^{13}C-NMR (150 MHz, CD_3OD) *δ*: 159.6 (C-3, 5), 158.3 (C-4′), 141.3 (C-1), 130.4 (C-1′), 129.4 (C-2′, 6′), 128.8 (C-8), 127.0 (C-7), 116.5 (C-3′, 5′), 105.8 (C-2, 6), 102.6 (C-4).

Trans-piceid (**38**). White powder; ESIMS (*m/z*): 413 $[M+Na]^+$; ^{1}H-NMR (400 MHz, CD_3OD) *δ*: 7.36 (2H, d, *J* = 8.4 Hz, H-2′,6′), 7.01 (1H, d, *J* = 16.0 Hz, H-8), 6.84 (1H, d, *J* = 16.0 Hz, H-7), 6.77 (2H, d, *J* = 6.4 Hz, H-3′, 5′), 6.74 (1H, t, *J* = 1.6 Hz, H-2), 6.60 (1H, t, *J* = 1.6 Hz, H-6), 6.44 (1H, t, *J* = 2.0 Hz, H-4), 4.82 (1H, d, *J* = 7.5Hz, H-1′); ^{13}C-NMR (100 MHz, CD_3OD) *δ*: 160.5 (C-3), 159.6 (C-5), 158.5 (C-4′), 141.4 (C-1), 130.3 (C-1′), 129.9 (C-8), 128.9 (C-2′, 6′), 126.7 (C-7), 116.5 (C-3′, 5′), 108.3

(C-6), 106.9 (C-2), 104.0 (C-4), 102.4(C-1″), 78.3 (C-3″), 78.1 (C-5″), 74.9 (C-2″), 71.5 (C-4″), 62.6 (C-6″).

Piceatannol (**39**). Pale yellow solid; 243 $[M-H]^-$; ^{1}H-NMR (400 MHz, CD_3OD) *δ*: 6.99 (1H, d, *J* = 2.0 Hz, H-2), 6.93 (1H, d, *J* = 16.0 Hz, H-8), 6.85 (1H, dd, *J* = 8.0, 2.0 Hz, H-6), 6.78 (1H, d, *J* = 16.0 Hz, H-7), 6.75 (1H, d, *J* = 1.6 Hz, H-5), 6.46 (2H, d, *J* = 2.0Hz, H-2′, 6′), 6.18 (1H, t, *J* = 2.0 Hz, H-4′); ^{13}C-NMR (100 MHz, CD_3OD) *δ*: 159.6 (C-3′, 5′), 146.5 (C-3), 146.5 (C-4), 141.3 (C-1′), 131.1 (C-1), 129.7 (C-7), 127.0 (C-8), 120.2 (C-6), 116.5 (C-2), 113.9 (C-5), 105.8 (C-2′, 6′), 102.7 (C-4′).

Trans-caffeic acid (**40**). Pale yellow powder; ^{1}H-NMR (500 MHz, CD_3OD) *δ*: 7.52 (1H, d, *J* = 15.5 Hz, H-7), 7.03 (1H, d, *J* = 2.0 Hz, H-2), 6.92 (1H, dd, *J* = 2.0, 8.0 Hz, H-6), 6.77 (1H, d, *J* = 8.5 Hz, H-5), 6.21 (1H, d, *J* = 16.0 Hz, H-8); ^{13}C-NMR (125 MHz, CD_3OD) *δ*: 171.1 (COOH), 149.4 (C-4), 147.0 (C-7), 146.8 (C-3), 127.8 (C-1), 122.8 (C-6), 116.5 (C-5), 115.5 (C-8), 115.1 (C-2).

5-O-Caffeoylshikimic acid (**41**). Pale yellow solid; $[\alpha]_D^{20}$ -97 (*c* 0.09, MeOH); ESIMS (*m/z*): 335 $[M-H]^-$, 359 $[M+Na]^+$; ^{1}H-NMR (600 MHz, CD_3OD) *δ*: 7.56 (1H, d, *J* = 15.6 Hz, H-9), 7.04 (1H, d, *J* = 2.4 Hz, H-2′), 6.95 (1H, dd, *J* = 7.8, 1.8 Hz, H-6′), 6.86 (1H, m, H-2), 6.77 (1H, d, *J* = 7.8 Hz, H-5′), 6.28 (1H, d, *J* = 15.6 Hz, H-8), 5.24 (1H, dt, H-5), 4.40 (1H, br. t, H-3), 3.91 (1H, dd, *J* = 7.2, 4.2 Hz, H-4), 2.85 (1H, dd, *J* = 18.2, 5.0 Hz, H-6b), 2.31 (1H, dd, *J* = 18.2, 5.2 Hz, H-6a); ^{13}C-NMR (150 MHz, CD_3OD) *δ*: 169.8 (COOH), 168.8 (C-7), 149.9 (C-4′), 147.5 (C-9), 147.0 (C-3′), 139.1(C-2), 130.5(C-1), 127.9 (C-1′), 123.2 (C-6′), 116.7 (C-5′), 115.4 (C-2′), 115.3 (C-8), 71.6 (C-5), 70.2 (C-4), 67.5 (C-3), 29.4 (C-6).

3-O-p-Coumaroylshikimic acid (**42**). Pale yellow solid; ESIMS (*m/z*): 319 $[M-H]^-$, 343 $[M+Na]^+$; ^{1}H-NMR (600 MHz, CD_3OD) *δ*: 7.63 (1H, d, *J* = 15.6 Hz, H-8′), 7.47 (2H, br. d, *J* = 9.0 Hz, H-3′, 5′), 6.81 (2H, br. d, *J* = 7.2 Hz, H-2′, 6′), 6.75 (1H, s, H-2), 6.35 (1H, d, *J* = 15.6 Hz, H-7′), 5.28 (1H, br. s, H-3), 4.40 (1H, m, H-5), 3.91 (1H, dd, *J* = 6.5, 4.0 Hz, H-4), 2.86 (1H, dd, *J* = 18.0, 4.8 Hz, H-6b), 2.32 (1H, dd, *J* = 18.0, 5.4 Hz, H-6a); ^{13}C-NMR (150 MHz, CD_3OD) *δ*: 168.6 (C-7, 9′), 161.3 (C-1′), 146.9 (C-7′), 134.5 (C-2), 133.6 (C-4′), 131.2 (C-3′/C-5′), 127.2 (C-1), 116.8 (C-3′/C-5′), 115.1 (C-8′), 71.4 (C-4), 70.1 (C-5), 67.4 (C-3), 29.4 (C-6).

(2S)-1,2-O-Di-trans-p-coumaroylglycerol (Smiglycerol) (**43**). Pale yellow powder; mp 183-185 ℃; $[\alpha]_D^{20}$ +164.7 (*c* 0.17, MeOH); CD (3.02 × 10^{-4} MeOH): Δε(nm) -6.68 (287), +5.04 (326); UV λ_{max} (MeOH): 210, 228, 310nm; IR υ_{max}^{KBr} cm^{-1}: 3448, 2926, 1686, 1634, 1516, 1438, 1342, 1287, 1150, 1052, 829; HR-ESI-MS *m/z*: 383.1133 $[M-H]^-$ (calcd. for $C_{21}H_{19}O_7$ 383.1131); 407.1103 $[M+Na]^+$ (calcd. for $C_{21}H_{20}O_7Na$ 407.1107); ^{1}H-NMR (600 MHz, DMSO-d_6) *δ*: 10.02 (2H, s, -OH), 7.57 (1H, d, *J* = 16.2 Hz, H-6), 7.541 (2H, d, *J* = 8.4 Hz, H-8, 12), 7.540 (1H, d, *J* = 15.6 Hz, H-3′), 7.53 (2H, d, *J* = 8.4 Hz, H-5′, 9′), 6.77 (2H, d, *J* = 8.4 Hz, H-9, 11), 6.76 (2H, d, *J* = 8.4 Hz, H-6′, 8′), 6.39 (1H, d, *J* = 16.2 Hz, H-5), 6.38 (1H, d, *J* = 15.6 Hz, H-2′), 5.11 (1H, m, H-2), 4.40 (1H, dd, *J* = 11.4, 3.0 Hz, H-1a), 4.25 (1H, dd, *J* = 12.0, 6.6 Hz, H-1b), 3.60 (2H, t, H-3a, H-3b); ^{13}C-NMR (150 MHz, DMSO- d_6) *δ*: 166.41 (C-1′), 166.19 (C-4), 159.92 (C-10), 159.89 (C-7′), 145.21 (C-6), 145.16 (C-3′), 130.44 (C-8,12),

130.40 (C-5′,9′), 125.02 (C-4′), 124.97 (C-7), 115.77 (C-9,11), 115.75 (C-6′,8′), 114.03 (C-5), 113.68 (C-2′), 72.13 (C-2), 62.54 (C-1), 59.67(C-3).

Juncusyl ester B (**44**). White powder; ESIMS (*m/z*): 237 $[M-H]^-$; ^{1}H-NMR (600 MHz, CD_3OD) δ: 7.67 (1H, d, J = 16.2 Hz, H-6), 7.46 (2H, d, J = 8.4 Hz, H-8, 12), 6.81 (2H, d, J = 9.0 Hz, H-9, 11), 6.37 (1H, d, J = 15.6 Hz, H-5), 4.99 (1H, m, H-2), 3.75 (4H, m, H-3), 3.75 (4H, m, H-1); ^{13}C-NMR (150 MHz, CD_3OD) δ: 168.9 (C-4), 161.2 (C-12), 146.6 (C-6), 131.1 (C-8), 131.1 (C-10), 127.2 (C-7), 116.8 (C-9), 116.8 (C-11), 115.4 (C-5), 76.6 (C-2), 61.7 (C-1), 61.7(C-3).

1-O-p-Coumaroylglycerol (**45**). Colorless solid; ESIMS (*m/z*): 237 $[M-H]^-$, 261 $[M+Na]^+$; ^{1}H-NMR (600 MHz, CD_3OD) δ: 7.66 (1H, d, J = 16.2 Hz, H-7′), 7.46 (2H, d, J = 9.5 Hz, H-2′, 6′), 6.81 (2H, d, J = 9.0 Hz, H-3′, 5′), 6.36 (1H, d, J = 15.6 Hz, H-8′), 4.26 (1H, dd, J = 11.4, 4.2 Hz, H-1a), 4.17 (1H, dd, J = 11.4, 6.6 Hz, H-1b), 3.90 (1H, J = 6.0 Hz, H-2), 3.61 (2H, d, J = 5.4 Hz, H-3); ^{13}C-NMR (150 MHz, CD_3OD) δ: 169.2 (C-9′), 161.3 (C-4′), 145.3 (C-7′), 131.2 (C-2′), 131.2 (C-6′), 127.2 (C-1′), 116.4 (C-3′), 116.4 (C-5′), 115.8 (C-8′), 71.3 (C-2), 66.6 (C-1), 64.1 (C-3).

Vanillin (**46**). Pale yellow oil; ^{1}H-NMR (500 MHz, CD_3OD) δ: 9.71 (1H, s, H-7), 7.42 (1H, s, H-2), 7.41 (1H, d, J = 7.0 Hz, H-6), 6.90 (1H, d, J = 7.5 Hz, H-5), 3.90 (3H, s, OCH_3); ^{13}C-NMR (125 MHz, CD_3OD) δ: 192.7 (C-7), 156.0 (C-4), 150.0 (C-3), 130.1 (C-1), 128.3 (C-6), 116.6 (C-5), 111.1 (C-2), 56.3 (OCH_3).

p-Hydroxy-benzaldehyde (**47**). Pale yellow powder; ^{1}H-NMR (500 MHz, CD_3OD) δ: 9.75 (1H, s, CHO), 7.76 (2H, d, J = 8.8 Hz, H-2, 6), 6.89 (2H, d, J = 8.4 Hz, H-3, 5); ^{13}C-NMR (125 MHz, CD_3OD) δ: 192.8 (CHO), 165.4 (C-4), 133.4 (C-2, 6), 130.2 (C-1), 116.9 (C-3, 5).

Acetovanillone (**48**). White powder; EIMS (*m/z*): 166 $[M]^+$; ^{1}H-NMR (600 MHz, DMSO-d_6) δ: 7.50 (1H, dd, J = 1.8, 8.4 Hz, H-6), 7.43 (1H, d, J = 1.8 Hz, H-2), 6.86 (1H, d, J = 8.4 Hz, H-5), 3.82 (3H, s, OCH_3), 2.50 (3H, s, H-8); ^{13}C-NMR (150 MHz, DMSO-d_6) δ: 196.0 (C-7), 153.8 (C-4), 147.5 (C-3), 128.8 (C-1), 123.4 (C-6), 114.9 (C-5), 111.1 (C-2), 55.6 (OCH_3), 26.2(C-8).

(+)-Scytalone (**49**). Pale yellow powder; $[\alpha]_D^{20}$ +10.6 (*c* 0.08, MeOH); ^{1}H-NMR (400 MHz, CD_3OD) δ: 6.22 (1H, br. s, H-5), 6.09 (1H, br. s, H-7), 4.25 (1H, m, H-3), 3.07 (1H, d, J = 16.0 Hz, H-4a), 2.84 (1H, d, J = 16.0 Hz, H-4b), 2.84 (1H, dd, J = 16.8, 3.2 Hz, H-2a), 2.61 (1H, dd, J = 17.2, 7.6 Hz, H-2b), ^{13}C-NMR (100 MHz, CD_3OD) δ: 202.4 (C-1), 166.7 (C-6), 166.5 (C-8), 146.0 (C-10), 111.7 (C-9), 109.4 (C-5), 101.7 (C-7), 66.9 (C-3), 47.4 (C-2), 39.2 (C-4).

Glucosyringic acid (**50**). White powder; ^{1}H-NMR (500 MHz, DMSO-d_6) δ: 12.87 (1H, s, COOH), 7.23 (2H, s, H-2, 6), 5.11 (1H, d, J = 7.5 Hz, H-1′), 3.79 (6H, s, 2 × OCH_3); ^{13}C-NMR (125 MHz, DMSO-d_6) δ: 166.9 (C-7), 152.2 (C-3, 5), 138.2 (C-4), 125.6 (C-1), 107.3 (C-2, 6), 101.9 (C-1′), 77.4 (C-5′), 76.6 (C-3′), 74.1 (C-2′), 69.8 (C-4′), 60.8 (C-6′), 56.3(2 × OCH_3).

Protocatechuic acid (**51**). White powder; EIMS *m/z*: 154 (M^+); ^{1}H-NMR (600 MHz, CD_3OD) δ: 7.43 (1H, s, H-2), 7.41 (1H, s, H-6), 6.79 (1H, d, J = 7.8 Hz, H-5); ^{13}C-NMR (150 MHz, CD_3OD) δ: 170.3 (COOH), 151.5 (C-4), 146.1 (C-3), 123.9 (C-6), 123.2 (C-3), 117.7 (C-2), 115.7(C-5).

3-Methoxygallic acid (**52**). White powder; ^{1}H-NMR (500 MHz, CD_3OD) δ: 7.18 (2H, s, H-2, 6), 3.86 (3H, s, OCH_3); ^{13}C-NMR (125 MHz, CD_3OD) δ: 170.2 (COOH), 149.1 (C-5), 146.2 (C-3), 140.4 (C-4), 122.0 (C-1), 112.2 (C-6), 106.3 (C-2), 56.6 (OCH_3).

Vanillic acid 1-O-β-D-glucopyranosyl ester (**53**). Pale yellow oil; $[\alpha]_D^{20}$ -16.5 (*c*0.07, MeOH); ^{1}H-NMR (500 MHz, CD_3OD) δ: 7.63 (1H, dd, *J* = 8.0, 2.0 Hz, H-6), 7.61 (1H, d, *J* = 2.0 Hz, H-2), 7.20 (1H, d, *J* = 8.5 Hz, H-5), 5.02 (1H, d, *J* = 7.5 Hz, GlcH-1′), 3.90 (3H, s, OCH_3), 3.68 (1H, dd, *J* = 12.0, 5.4 Hz, GlcH-6′), 3.54-3.38 (4H, m, GlcH-2′, 3′, 4′, 5′); ^{13}C-NMR (125 MHz, CD_3OD) δ: 169.5 (C-7), 152.0 (C-4), 150.4 (C-3), 126.0 (C-6), 124.8 (C-1), 116.4 (C-5), 114.4 (C-2), 101.9 (C-1′), 77.9 (C-5′), 78.3 (C-3′), 74.8 (C-2′), 71.3 (C-4′), 62.4 (C-6′), 56.6 (OCH_3).

Hydroxytyrosol (**54**). Pale yellow solid; ^{1}H-NMR (600 MHz, CD_3OD) δ: 6.67 (1H, d, *J* = 8.4Hz, H-5), 6.65 (1H, d, *J* = 2.4 Hz, H-2), 6.52 (1H, dd, *J* = 8.4, 2.4 Hz, H-6), 3.67 (2H, t, *J* = 7.2 Hz, H-2'), 2.66 (2H, t, *J* = 7.2 Hz, H-1'); ^{13}C-NMR (150 MHz, CD_3OD) δ: 146.1 (C-3), 144.6 (C-4), 131.8 (C-1), 121.2 (C-6), 117.1 (C-5), 116.3 (C-2), 64.6 (C-2′), 39.7 (C-1′).

Acetyl-11-keto-β-boswellic acid (**55**). Pale yellow solid; EIMS *m/z*: 512 (4) $[M]^+$, 232 (100), 408 (53), 273 (45), 393 (28); ^{1}H-NMR (500 MHz, $CDCl_3$) δ: 5.56 (1H, s, H-12), 5.31 (1H, t, *J* = 2.5 Hz, H-3), 2.42 (1H, s, H-9), 2.09 (3H, s, CH_3CO), 1.35 (3H, s, CH_3-23), 1.24 (3H, s, CH_3-27), 1.20 (3H, s, CH_3-26), 1.15 (3H, s, CH_3-25), 0.95 (3H, s, CH_3-30), 0.83 (3H, s, CH_3-28), 0.81 (3H, d, *J* = 6.5 Hz, CH_3-29); ^{13}C-NMR (125 MHz, CD_3OD) δ: 201.6 (C-11), 179.6 (C-24), 172.3 ($COCH_3$), 167.9 (C-13), 131.2 (C-12), 74.9 (C-3), 61.7 (C-9), 60.5 (C-18), 51.5 (C-5), 46.4 (C-4), 45.1 (C-14), 42.0 (C-8), 40.6 (C-22), 40.5 (C-19), 40.4 (C-20), 38.6 (C-10), 35.8 (C-1), 35.1 (C-17), 33.9 (C-7), 30.8 (C-21), 29.4 (C-28), 28.6 (C-15), 28.3 (C-16), 24.5 (C-23), 24.4 (C-2), 21.5 ($COCH_3$), 21.1 (C-27), 20.8 (C-30), 20.1 (C-6), 18.9 (C-26), 17.8 (C-29), 14.4 (C-25).

Stigmasterol (**56**). White powder; ^{1}H-NMR (500 MHz, $CDCl_3$) δ: 5.35 (1H, br d, *J* = 5.5 Hz, H-6), 5.15 (1H, dd, *J* = 8.5, 15.5 Hz, H-22), 5.02 (1H, dd, *J* = 8.5 Hz, H-23), 3.52 (1H, m, H-3), 0.93 (3H, d, *J* = 6.0 Hz, H-21), 0.86 (3H, t, *J* = 7.0 Hz, H-29), 0.84 (3H, d, *J* = 7.5 Hz, H-26), 0.82 (3H, d, *J* = 7.5 Hz, H-27), 0.80 (3H, s, H-19), 0.68 (3H, s, H-18); ^{13}C-NMR (125 MHz, $CDCl_3$) δ: 140.8 (C-5), 138.3 (C-22), 129.3 (C-23), 121.7 (C-6), 71.8 (C-3), 56.9 (C-17), 55.9 (C-14), 51.2 (C-24), 50.2 (C-9), 42.2 (C-13), 40.5 (C-20), 39.7 (C-4), 39.7 (C-12), 36.5 (C-10), 33.2 (C-25), 31.9 (C-2), 31.9 (C-7), 31.9 (C-8), 31.7 (C-1), 28.9 (C-16), 25.4 (C-28), 24.4 (C-15), 21.2 (C-11), 21.1 (C-21), 21.1 (C-27), 19.4 (C-19), 19.0 (C-26), 12.2 (C-29), 12.0 (C-18).

β-Sitosterol (**57**). White powder; ^{1}H-NMR (400 MHz, $CDCl_3$) δ: 5.35 (1H, br d, *J* = 5.2 Hz, H-6), 3.55 (1H, m, H-3), 1.00 (3H, s, 19-CH_3), 0.93 (3H, d, *J* = 8.0 Hz, 21-CH_3), 0.91 (3H, d, *J* = 8.0 Hz, 29-CH_3), 0.86 (3H, d, *J* = 7.6 Hz, 26-CH_3), 0.84 (3H, d, *J* = 7.6 Hz, 27-CH_3), 0.79 (3H, s, 18-CH_3); ^{13}C-NMR (100 MHz, $CDCl_3$) δ: 140.8 (C-5), 121.7 (C-6), 71.8 (C-3), 56.8 (C-14), 56.1 (C-17), 50.1 (C-9), 45.8 (C-4), 42.3 (C-13), 39.7 (C-12), 37.3 (C-1), 36.5 (C-10), 36.1 (C-20), 34.0 (C-22), 31.9 (C-8), 31.7 (C-7), 29.2 (C-2), 28.2 (C-25), 26.1 (C-15), 24.3 (C-23), 23.1 (C-28), 21.1 (C-11), 19.8 (C-26), 19.4 (C-19), 19.0 (C-21), 18.8 (C-27), 12.0 (C-29), 11.9 (C-18).

Daucosterol (**58**). White powder; ^{1}H-NMR (400 MHz, C_5D_5N) δ: 5.34 (1H, m, H-6), 5.06 (1H, d, J = 7.6 Hz, Glc H-1′); ^{13}C-NMR (100 MHz, C_5D_5N) δ: 140.9 (C-5), 121.9 (C-6), 102.6 (Glc C-1′), 78.6 (C-3), 78.5 (Glc C-3′), 78.1 (Glc C-5′), 75.3 (Glc C-2′), 71.7 (Glc C-4′), 62.9 (Glc C-6′), 56.8 (C-14), 56.3 (C-17), 50.4 (C-9), 46.1 (C-24), 42.5 (C-13), 40.0 (C-4), 39.4 (C-12), 37.5 (C-1), 36.9(C-20), 36.4 (C-10), 34.2 (C-22), 32.2 (C-8), 32.1 (C-7), 30.3 (C-2), 29.5 (C-25), 28.6 (C-16), 26.4 (C-23), 24.5 (C-15), 23.4 (C-28), 21.3 (C-11), 20.0 (C-27), 19.4 (C-19), 19.2 (C-26), 19.0 (C-21), 12.2 (C-18), 12.0 (C-29).

Smilagenin (**59**). White powder; ESIMS (*m/z*): 339 $[M-H]^-$, 363$[M+Na]^+$; ^{1}H-NMR (500 MHz, CD_3OD) δ: 8.09 (1H, s, H-2), 6.32 (1H, d, J = 2.0 Hz, H-8), 6.20 (1H, d, J = 2.0 Hz, H-6), 5.31 (1H, d, J = 1.5 Hz, H-1′), 3.82 (1H, dd, J = 3.5, 9.5 Hz, H-2′), 3.76 (1H, dq, J = 9.0, 6.0 Hz, H-5′), 3.44 (1H, dd, J = 1.5, 9.5 Hz, H-3′), 3.30 (1H, s, H-4′), 1.27 (3H, d, J = 6.0 Hz, H-6′); ^{13}C-NMR (125 MHz, CD_3OD) δ: 178.7 (C-4), 166.5 (C-7), 163.5 (C-5), 159.3 (C-9), 147.9 (C-2), 140.4 (C-3), 106.4 (C-10), 102.1 (C-1′), 100.1 (C-6), 95.0 (C-8), 73.5 (C-4′), 71.9 (C-3′), 71.6 (C-2′), 71.2 (C-5′), 18.0(C-6′).

5-Hydroxymaltol (**60**). White powder; EIMS *m/z*: 142 $[M]^+$; ^{1}H-NMR (600 MHz, CD_3OD) δ: 7.85 (1H, s, H-2), 2.32 (3H, s, CH_3); ^{13}C-NMR (150 MHz, CD_3OD) δ: 170.3 (C-4), 151.8 (C-3), 145.8 (C-6), 142.9 (C-5), 140.4 (C-2), 14.5(CH_3).

5-Hydroxyuridine (**61**). Pale yellow solid; ^{1}H-NMR (500 MHz, DMSO-d_6) δ: 8.11 (1H, d, J = 8.5 Hz, H-6), 5.90 (1H, d, J = 6.0 Hz, H-1′), 5.74 (1H, d, J = 6.5 Hz, H-5), 5.40 (1H, d, J = 5.6 Hz), 5.13 (1H, d, J = 4.8 Hz), 5.11 (1H, d, J = 4.8 Hz), 4.55 (1H, d, J = 5.0 Hz, H-2′), 4.14 (1H, t, J = 8.5 Hz, H-3′), 3.97 (1H, d, J = 4.0 Hz, H-4′), 3.67 (1H, m, H-5′a), 3.56 (1H, m, H-5′e); ^{13}C-NMR (125 MHz, DMSO-d_6) δ: 158.7 (C-4), 149.1 (C-2), 140.9 (C-6), 101.7 (C-5), 87.8 (C-1′), 85.9 (C-4′), 73.8 (C-3′), 70.4 (C-2′), 61.3 (C-5′).

2-Methyl butanedioic acid-4-ethyl ester (**62**). Pale yellow oil; ^{1}H-NMR (500 MHz, CD_3OD) δ: 4.11 (2H, dd, J = 8.5, 5.5 Hz, H-6), 2.85 (1H, m, H-2), 2.64 (1H, dd, J = 17.0, 8.5 Hz, H-3b), 2.41 (1H, dd, J = 16.5, 5.5 Hz, H-3a), 1.23 (3H, t, J = 7.0 Hz, H-7), 1.20 (3H, d, J = 7.5 Hz, H-5); ^{13}C-NMR (125 MHz, CD_3OD) δ: 179.1 (C-1), 173.8 (C-4), 61.6 (C-6), 38.7 (C-3), 37.5 (C-2), 17.5 (C-5), 14.5(C-7).

Isoselachoceric acid (**63**). Pale yellow powder; EIMS *m/z* (%): 368 (M^+); ^{1}H-NMR (400 MHz, $CDCl_3$) δ: 2.36 (2H, t, J = 7.2 Hz, H-2), 1.63 (2H, m, H-3), 1.27 (19 × CH_2, 38H, br. s), 0.87 (3H, t, J = 7.0 Hz, H-24).

Figure S1. Structures of the investigated flavan-3-ols (**1**, **20**–**22**).

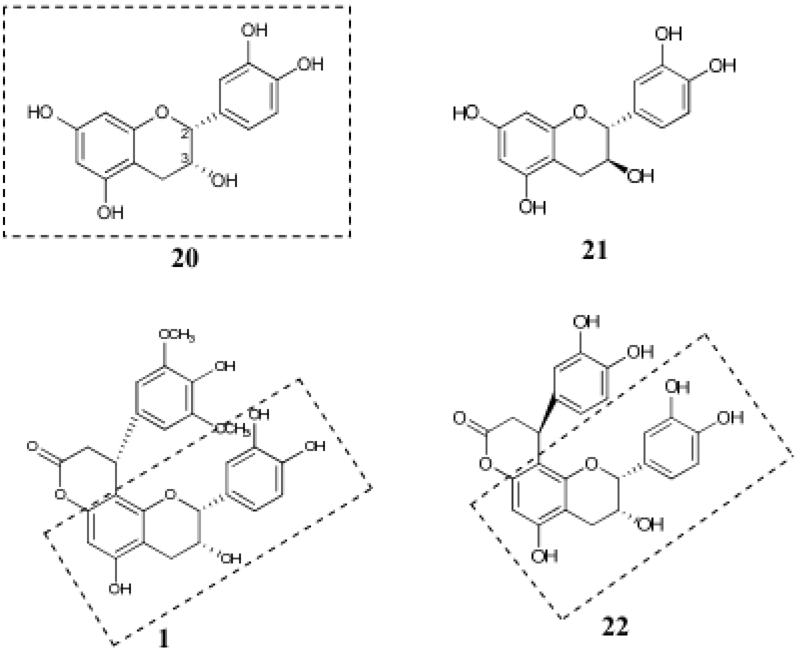

Table S1. MIC values of investigated flavan-3-ols (**1**, **20**–**22**) against the microorganisms tested.

Compound	Microorganisms (MICs, mM)
**1**	EC, PA, KP (0.604); MRSA, SA, EF (0.302); CA (0.146)
**20**	MRSA, EF (1.03); SA (0.517)
**21**	>2.07
**22**	PA (0.663); MRSA, SA (0.0801); EF, CA (0.160)

Figure S2. Flavanone skeletons of compounds **7**, **11**–**14**.

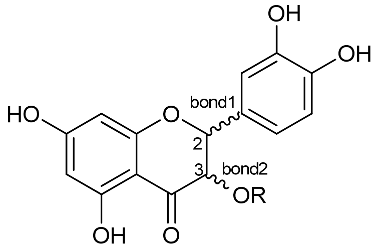

Table S2. MIC values of investigated flavanones (**7**, **11**–**14**) against the microorganisms tested.

Compound	R	Bond 1	Bond 2	Microorganisms (MICs, mM)
**7**	H	''''''''||||	◀	>1.97
**11**	Rha	''''''''||||	''''''''||||	>1.33
**12**	Rha	''''''''||||	◀	CA (0.666)
**13**	Rha	◀	''''''''||||	MRSA(1.33)
**14**	Rha	◀	◀	SA (0.666); EC, PA, KP, MRSA, EF, CA (1.33)

Figure S3. Flavanone skeletons of compounds **9**, **15–17**.

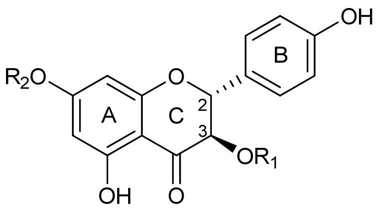

Table S3. MIC values of investigated flavanones (**9**, **15–17**) against the microorganisms tested.

Compound	R_1	R_2	Microorganisms (MICs, mM)
**9**	H	H	MRSA, SA (2.10)
**15**	Rha	H	>1.38
**16**	Glc	H	>1.33
**17**	H	Glc	MRSA, SA, EF (1.33)

Figure S4. Stibene skeletons of compounds **37** and **38**.

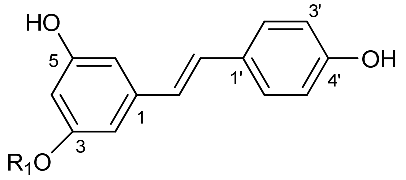

Figure S5. Stibene skeletons of compounds **4** and **39**.

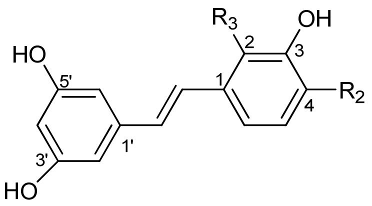

Table S4. MIC values of investigated stibenes (**4**, **37–39**) against the microorganisms tested.

Compound	R_1	R_2	R_3	Microorganisms (MICs, mM)
**37**	H	-	-	MRSA, EF (0.159); SA (0.0794); CA (0.657)
**38**	Glc	-	-	MRSA, SA (0.768); EF (1.54)
**4**	-	H	OH	EC, PA, KP (1.64); MRSA (0.409); SA (0.205); EF, CA (0.819)
**39**	-	OH	H	EC, PA, KP (1.64); MRSA (0.409); SA (0.205); CA (0.819)

EC, *Escherichia coli*; PA, *Pseudomonas aeruginosa* PA01; KP, *Kiebsiella pneumonia*(clinical isolate); MRSA, methicillin-resistant *Staphylococcus aureus*(clinical isolate); SA, *Staphylococcus aureus* ATCC6538; EF, *Enterococcus faecalis*; CA, *Canidia albicans* SC5314.
